# Identifying TNF and IL6 as potential hub genes and targeted drugs associated with scleritis: A bio-informative report

**DOI:** 10.3389/fimmu.2023.1098140

**Published:** 2023-03-31

**Authors:** Feiyue Yan, Yizong Liu, Tianlu Zhang, Yin Shen

**Affiliations:** ^1^ Eye Center, Renmin Hospital of Wuhan University, Wuhan, China; ^2^ Frontier Science Center of Immunology and Metabolism, Medical Research Institute, Wuhan University, Wuhan, China

**Keywords:** scleritis, PPI network, hub genes, drug targets, TNF, IL6

## Abstract

**Background:**

Scleritis is a serious inflammatory eye disease that can lead to blindness. The etiology and pathogenesis of scleritis remain unclear, and increasing evidence indicates that some specific genes and proteins are involved. This study aimed to identify pivotal genes and drug targets for scleritis, thus providing new directions for the treatment of this disease.

**Methods:**

We screened candidate genes and proteins associated with scleritis by text-mining the PubMed database using Python, and assessed their functions by using the DAVID database. Gene Ontology (GO) and Kyoto Encyclopedia of Genes and Genomes (KEGG) analyses were used to identify the functional enrichment of these genes and proteins. Then, the hub genes were identified with CytoHubba and assessed by protein-protein interaction (PPI) network analysis. And the serum from patients with active scleritis and healthy subjects were used for the validation of hub genes. Finally, the DGIdb database was used to predict targeted drugs for the hub genes for treating scleritis.

**Results:**

A total of 56 genes and proteins were found to be linked to scleritis, and 65 significantly altered pathways were identified in the KEGG analysis (FDR < 0.05). Most of the top five pathways involved the categories “Rheumatoid arthritis,” “Inflammatory bowel disease”, “Type I diabetes mellitus,” and “Graft-versus-host disease”. TNF and IL6 were considered to be the top 2 hub genes through CytoHubba. Based on our serum samples, hub genes are expressed at high levels in active scleritis. Five scleritis-targeting drugs were found among 88 identified drugs.

**Conclusions:**

This study provides key genes and drug targets related to scleritis through bioinformatics analysis. TNF and IL6 are considered key mediators and possible drug targets of scleritis. Five drug candidates may play an important role in the diagnosis and treatment of scleritis in the future, which is worthy of the further experimental and clinical study.

## Introduction

Scleritis is an inflammatory condition causing sharp pain in the sclera, which in severe cases may spread to the cornea and uvea (1). According to the site of the lesion, it can be divided into anterior scleritis, posterior scleritis and panscleritis. It usually occurs in people between the ages of 40 and 60, with females affected more commonly, and both eyes are involved in more than 50% of patients. Without timely treatment, ocular structure and function may be affected, sometimes even leading to blindness (2). At present, the etiology and pathogenesis of scleritis have not been fully clarified. 4% to 10% of all reported cases are caused by infections with bacteria, such as Treponema pallidum, or viruses, such as varicella-zoster virus (3, 4). It has also been associated with autoimmune diseases such as rheumatoid arthritis (RA) and systemic lupus erythematosus (5, 6), as well as metabolic diseases like gout (7). Malignancy, surgical induction, or medication side effects have been suggested as possible causes of scleritis (8, 9). In addition, the diagnosis of scleritis is complex and is mainly based on clinical manifestations and ophthalmic examinations. For the posterior scleritis, ultrasound or ocular imaging tests are also required to confirm the diagnosis (10, 11).

The treatment of scleritis mainly involves suppressing inflammation and reducing tissue damage. Currently, the main clinical treatment for infectious scleritis is based on the primary cause of infection. For noninfectious scleritis, the major therapeutic interventions are systemic glucocorticoids, nonsteroidal anti-inflammatory drugs (NSAIDs) and immunosuppressants (12). However, long-term use of these drugs may cause side effects such as hyperglycemia, gastrointestinal lesions, and severe liver and kidney toxicity. It has been demonstrated that biological therapies that target specific mediators and cells of the immune system and inflammatory response can be more specific and selective for precise treatment (13, 14). Therefore, it is necessary to further understand the pathogenesis of scleritis and screen new and more effective target genes and key drugs to control symptoms and improve prognosis.

Although the lack of specific and reproducible animal models of scleritis has hindered further research into the pathogenesis and treatment of this disease, accumulating evidence has proven that some specific genes and proteins are involved in the occurrence and development of scleritis. In this study, we identified genes and proteins associated with scleritis from the literature, analyzed them through a bioinformatics approach, explored the pathogenesis of scleritis and crucial drug targets for this disease, and provided a new direction for treatment.

## Materials and methods

### Text-mining of scleritis manuscripts

We comprehensively reviewed the literature related to the keyword “scleritis” in any language published in the PubMed database from 1910 to February 2022, using the computer programming language Python. Then, we performed text mining of common words from the abstract. These publications were summarized in a word cloud, with noninformative common English terms curated and removed manually (15).

### Identifying target genes

According to similar methods previously used by others (16, 17), we manually collected candidate genes and proteins linked to scleritis. The database was eligible if the study included comparisons of gene or protein expression levels between scleritis patients and normal controls. The main exclusion criteria included: (i) meta-analyses and reviews; (ii) experimental studies conducted only in animal models; (iii) other articles not related to the topic.

### Gene function analysis

Gene function was analyzed using the Database for Annotation, Visualization and Integrated Discovery (DAVID), version 2021 (https://david.ncifcrf.gov) (18). According to the Gene Ontology (GO) database, an ontology database that describes the functions of all genes, candidate genes and proteins were categorized as cellular component (CC), molecular function (MF) or biological process (BP) (19). CC indicates the cellular location where a particular gene product performs its molecular function. MF refers to the molecular-level activity that a particular gene product performs, such as “binding activity” or “protein activity”. BP terms describe a series of events that are accomplished by one or more organized sets of molecular functions, for instance, “cellular physiological processes” and “signaling”. Following the upload of the gene list to the website, we performed the analysis of the above three categories and selected terms with FDR<0.05.

### KEGG pathway analysis

Enriched candidate genes were assessed with Kyoto Encyclopedia of Genes and Genomes (KEGG) pathway analysis on the DAVID platform, and pathways including at least three genes with an FDR<0.05 were selected. KEGG is an online database that systematically analyzes gene functions from the perspective of gene and molecular networks. The KEGG database contains maps of the most known metabolic pathways and regulatory pathways. Graphical information rather than complex text is used in KEGG to introduce each metabolic pathway and its relationship to others (20).

### Protein-protein interaction (PPI) network analysis

PPIs were analyzed using candidate genes and proteins in the Search Tool for the Retrieval of Interacting Genes (STRING), version 11.5 (https://cn.string-db.org/). STRING is a biological database of known and predicted PPIs in molecular biology (21). The network type used was “Full STRING Type”, “Required score=0.4”, and “FDR stringency=5%”. PPI network visualization was performed with Cytoscape software (version 3.8.1). Cytoscape consists of a network of nodes and edges. Each node can be a gene, miRNA or protein, and the edges represent interactions between them. The candidate genes or proteins were clustered and ranked using molecular complex detection (MCODE), a plugin for Cytoscape (22), with parameters set to degree cutoff=2, node score cutoff = 0.2, k-core=2, and max depth=100.

### Hub genes identification and validation *in vitro*

CytoHubba, an app of Cytoscape, which assigns a value to each gene through a topological network algorithm, sequentially identifies hub genes and subnetworks, and ranks nodes according to their properties in the network (23). The nodes were ranked using the five algorithms provided by CytoHubba, including the maximum neighborhood component (MNC), maximal clique centrality (MCC), degree, edge percolated component (EPC), and closeness. To improve the accuracy of the prediction results, these five methods were examined separately and the top ten significant nodes derived from each were used as intersection sets to identify the hub genes. The STRING online database was used for the coexpression analysis of hub genes. A heatmap of the coexpression scores of these genes was created from RNA expression patterns, and protein co-regulation provided by ProteomeHD (https://www.proteomehd.net/index). Five patients with active scleritis and five healthy control adults were recruited from the Eye Center, Renmin Hospital of Wuhan University (2021–2023), and their serum samples were collected for enzyme-linked immunosorbent assay (ELISA) validation. The inclusion criteria for patients were as follow: (i) the presence of swelling and edema of the scleral tissue and congestion of the deeper episcleral vessels, and diagnosed by an experienced ophthalmologist as scleritis (24); (ii) no other ocular disease (glaucoma, high myopia, strabismus, optic atrophy, etc.). The exclusion criteria were as follow: (i) history of eye surgery and ocular trauma; (ii) uncertain diagnoses. General information about the participants was provided in **Supplementary Table S1**. The study protocol was approved by the Research Ethics Committee of the Renmin Hospital of Wuhan University (WDRY2019-K032), and informed consent was obtained from all participants and/or minor(s)’ legal guardian/next of kin.

### ELISA

Protein expression in serum samples was determined by ELISA. The serum levels of TNF, IL6, IFNγ, IL1β, ICAM1, IL17A, MMP9, IL4, IL12, TGFβ (Thermo Fisher Scientific, US), and HMGB1 (Arigo Biolaboratories, China) were determined by ELISA following the manufacturer instructions. The statistical analyses were carried out using GraphPad Prism 8.0. All experiments were performed by a two-tailed Student’s t-test. *P*<0.05 was considered significant.

### Prediction of potential drugs for key genes

The Drug-Gene Interaction Database (DGIdb, version 4.2.0, http://www.dgidb.org/) provides an interface to search for drug-gene interactions and drug-ready genomes by leveraging existing resources. Potential drugs that interact with the top two genes above were found using DGIdb (25). Unique matches and selected interaction scores were used to evaluate the strength of drug-gene interactions.

## Results

### Overview of research findings

**Figure 1:**
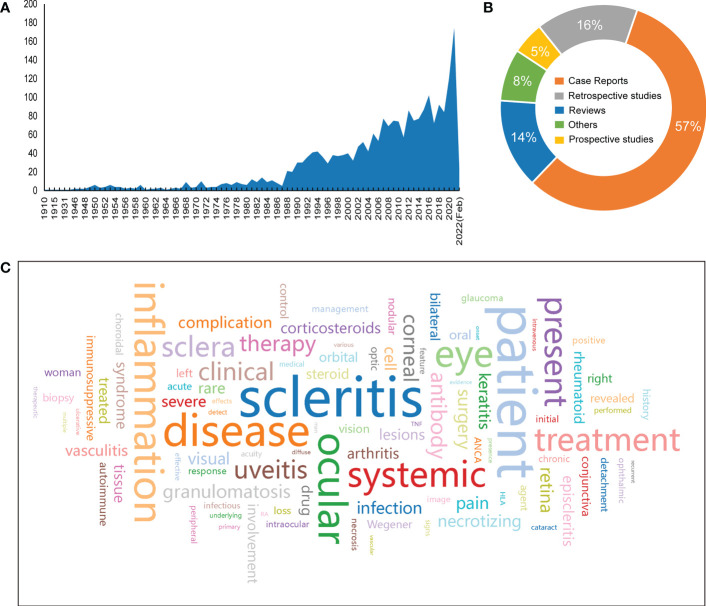
Overview of research findings. **(A)**Number of articles published from 1910 to February 2022. **(B)** Percentage of article categories. **(C)** Word cloud of the 100 most frequently used words in the literature abstract (the larger the font size, the higher the frequency).

The search results indicate that the number of case reports and articles related to scleritis has generally increased since the publication of the first article on scleritis in 1910, with a more pronounced trend from 1988 onward (**Figure 1A**). Up to February 2022, a total of 2290 publications on scleritis were identified in PubMed, with case reports accounting for the largest share of 57% (**Figure 1B**). Retrospective studies were more common than prospective studies in terms of study type.

To identify the most studied topics or areas of interest in the current literature on scleritis, we analyzed the frequency of biological terms used in the abstracts of the retrieved literature and created a word cloud map of the top 100 words (**Figure 1C**). Notably, the most common terminology was “scleritis, patient, disease, inflammation, ocular, eye, systemic, treatment, present, sclera, uveitis, clinical, therapy, antibody, corneal, granulomatosis”, which generally shows that most of the articles focus on the diagnosis and treatment of scleral infection. Terms such as “patient, disease, ocular, eye, retina, pain” are often used in abstracts as an introduction to facts rather than the subject of the article. Other terms, such as “systemic, autoimmune, rheumatoid”, are used to study scleritis associated with autoimmune disease, which provides a partial basis for finding potential targets for scleritis pathogenesis.

### Generating the required proteins and genes

**Figure 2:**
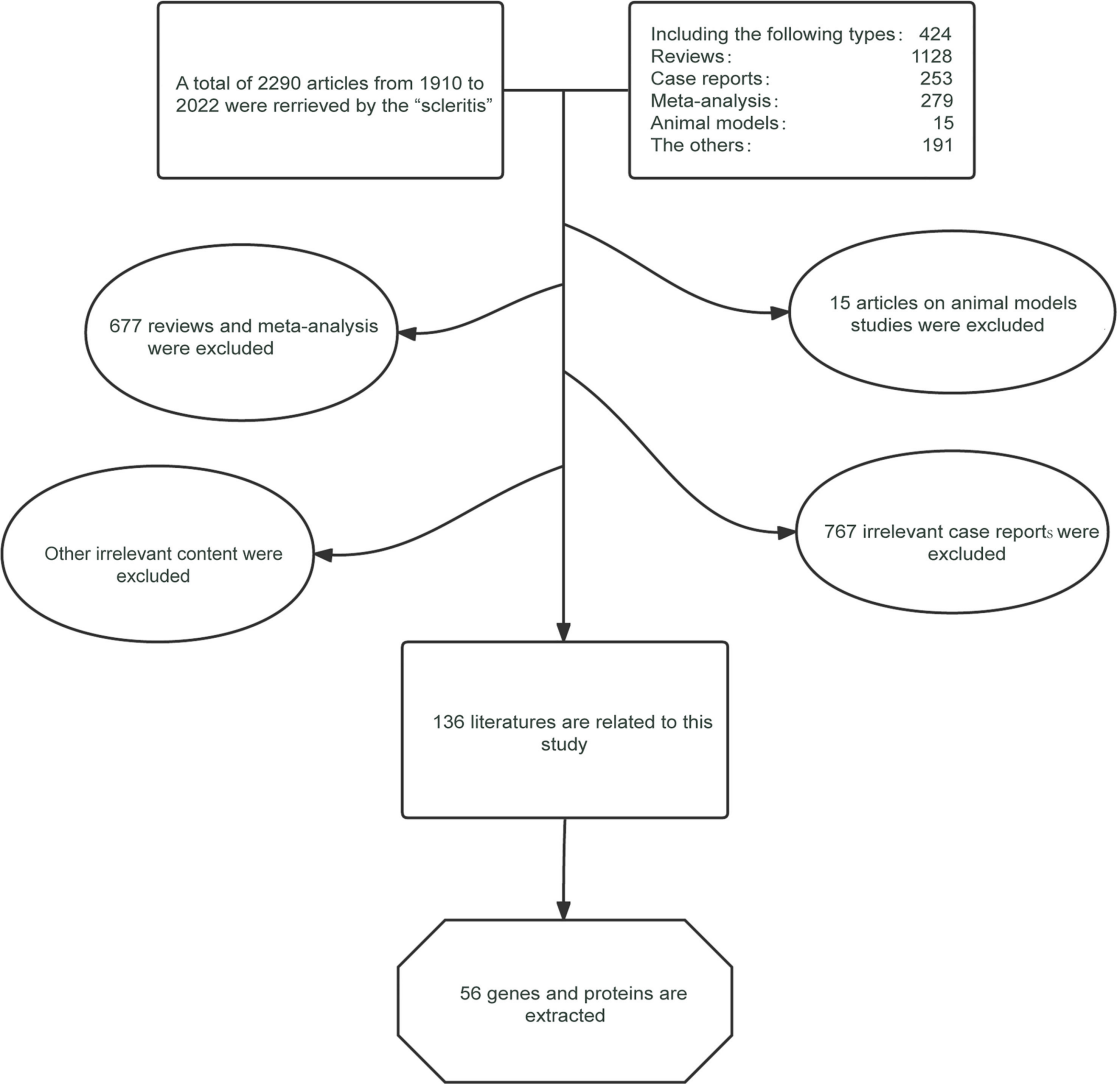
Flow chart for the selection of candidate genes and proteins.

We screened 2290 articles from PubMed and found that 136 were eligible (**Figure 2**). Fifty-six genes and proteins related to scleritis were identified based on the inclusion and exclusion criteria (**Table 1**). According to previous studies, these screened genes were expressed differently in patients with scleritis and healthy individuals. A total of 56 genes are listed in **Supplementary Table 2** with data sources, regulation of pathogenesis, and classification by etiology and location.

**Table 1:** Scleritis-associated genes selected from eligible articles.

Number	Gene	Gene ID	Description	Aliases	PMID
	Symbol				
1	ACE2	59272	angiotensin converting enzyme 2	ACEH	34351874
**2**	CD3G	917	CD3 gamma subunit of T-cell receptor complex	CD3-GAMMA, CD3GAMMA, IMD17, T3G	18303163
**3**	CD4	920	CD4 molecule	CD4mut, IMD79, OKT4D	18303163/ 32615955
**4**	CD68	968	CD68 molecule	GP110, LAMP4, SCARD1	18303163
**5**	CRP	1401	C-reactive protein	PTX1	35866779/ 23030356
**6**	CTLA4	1493	cytotoxic T-lymphocyte associated protein 4	ALPS5, CD, CD152, CELIAC3, CTLA-4, GRD4, GSE, IDDM12	30921471
**7**	FAS	355	Fas cell surface death receptor	ALPS1A, APO-1, APT1, CD95, FAS1, FASTM, TNFRSF6	19412867
**8**	FASLG	356	Fas ligand	ALPS1B, APT1LG1, APTL, CD178, CD95-L, CD95L, FASL, TNFSF6, TNLG1A	19412867
**9**	FCGBP	8857	Fc gamma binding protein	FC(GAMMA)BP	14722460
**10**	GZMB	3002	granzyme B	C11, CCPI, CGL-1, CGL1, CSP-B, CSPB, CTLA1, CTSGL1, HLP, SECT	25750517
**11**	HLA-A	3105	major histocompatibility complex, class I, A	HLAA	11339599
**12**	HLA-B	3106	major histocompatibility complex, class I, B	AS, B-4901, HLAB	17579295/ 27428361
**13**	HLA-C	3107	major histocompatibility complex, class I, C	D6S204, HLA-JY3, HLAC, HLC-C, MHC, PSORS1	9822977
**14**	HLA-DQA1	3117	major histocompatibility complex, class II, DQ alpha 1	CELIAC1, DQ-A1, DQA1, HLA-DQA,	9822977
**15**	HLA-DQB1	3119	major histocompatibility complex, class II, DQ beta 1	HLA-DQB, IDDM1	9124099
**16**	HLA-DRB1	3123	major histocompatibility complex, class II, DR beta 1	DRB1, HLA-DR1B	9124099
**17**	HLA-DRB4	3126	major histocompatibility complex, class II, DR beta 4	DR4, DRB4, HLA-DR4B	9822977
**18**	ICAM1	3383	intercellular adhesion molecule 1	BB2, CD54, P3.58	19412867
**19**	IFNγ	3458	interferon gamma	IFG, IFI, IMD69	17496900
**20**	IGAN1	60498	IgA nephropathy	IGAN	36191648
**21**	IL17	3605	interleukin 17A	CTLA8; IL-17; ILA17; CTLA-8; IL-17A	17496900
**22**	IL18	3606	interleukin 18	IGIF, IL-18, IL-1g, IL1F4	20505198
**23**	IL1β	3553	interleukin 1 beta	IL1-BETA, IL1F2, IL1beta	27442322
**24**	IL1RA	3552	interleukin 1 alpha	IL-1 alpha, IL-1A, IL1F1	17449489/ 28537473
**25**	IL2	3558	interleukin 2	IL-2, TCGF, lymphokine	17496900
**26**	IL22	50616	interleukin 22	IL-D110, IL-TIF, ILTIF, TIFIL-23, TIFa, zcyto18	27009382
**27**	IL27	246778	interleukin 27	IL-27A, IL27p28, IL30, p28	17496900
**28**	IL6	3569	interleukin 6	BSF-2, BSF2, CDF, HGF, HSF, IFN-beta-2, IFNB2	27010181/ 34615818
**29**	ITGAL	3683	integrin subunit alpha L	CD11A, LFA1A	9823349
**30**	ITGB2	3689	integrin subunit beta 2	CD18, LAD, LCAMB, LFA-1, MAC-1, MF17, MFI7	19412867
**31**	KRT19	3880	keratin 19	CK19, K19, K1CS	29794824
**32**	MEFV	4210	MEFV innate immuity regulator, pyrin	FMF; MEF; PAAND; TRIM20	32231554
**33**	MMP1	4312	matrix metallopeptidase 1	CLG, CLGN	9645358
**34**	MMP10	4319	matrix metallopeptidase 10	SL-2, STMY2	27478294
**35**	MMP13	4322	matrix metallopeptidase 13	CLG3, MANDP1, MDST, MMP-13	27478294
**36**	MMP2	4313	matrix metallopeptidase 2	CLG4, CLG4A, MMP-II, MONA, TBE-1	9033278
**37**	MMP3	4314	matrix metallopeptidase 3	CHDS6, SL-1, STMY, STMY1, STR1	22823240/ 9645358
**38**	MMP8	4317	matrix metallopeptidase 8	CLG1, HNC, PMNL-CL	15086386
**39**	MMP9	4318	matrix metallopeptidase 9	CLG4B, GELB, MANDP2	22823240/ 9645358
**40**	MPO	4353	myeloperoxidase	–	17401624/ 31941745
**41**	MS4A1	931	membrane spanning 4-domains A1	B1, Bp35, CD20, CVID5, FMC7, LEU-16, S7	15958765
**42**	MT-CO1	4512	mitochondrially encoded cytochrome c oxidase I	COI, MTCO1, COX1	22642498
**43**	MT-CO2	4513	mitochondrially encoded cytochrome c oxidase II	COII, MTCO2, COX2	15953441
**44**	PDGFRA	5156	platelet derived growth factor receptor alpha	CD140A, PDGFR-2	20505198
**45**	PDGFRB	5159	platelet derived growth factor receptor beta	CD140B, IBGC4, IMF1, JTK12, KOGS, PDGFR, PDGFR1, PENTT	20505198
**46**	PRTN3	5657	proteinase 3	ACPA, AGP7, C-ANCA, CANCA, MBN, MBT, NP-4, NP4, P29, PR3	26507398/ 29042820
**47**	PTPN22	26191	protein tyrosine phosphatase non-receptor type 22	LYP, LYP1, LYP2, PEP, PTPN22.5, PTPN22.6, PTPN8	30921471
**48**	SOCS1	8651	suppressor of cytokine signaling 1	AISIMD, CIS1, CISH1, JAB, SOCS-1, SSI-1, SSI1, TIP-3, TIP3	21778271
**49**	SSB	6741	small RNA binding exonuclease protection factor La	LARP3, La, La/SSB	35914299
**50**	STAT1	6772	signal transducer and activator of transcription 1	CANDF7, IMD31A, IMD31B, IMD31C, ISGF-3, STAT91	17496900
**51**	THBD	7056	thrombomodulin	AHUS6, BDCA-3, BDCA3, CD141, THPH12, THRM, TM	20505198
**52**	TIA1	7072	T-cell intracellular antigen 1	TIA-1,	17893556
**53**	TIMP1	7076	TIMP metallopeptidase inhibitor 1	CLGI, EPA, EPO, HCI, TIMP	9033278
**54**	TNF	7124	tumor necrosis factor	DIF, TNF-alpha, TNFA, TNFSF2, TNLG1F	33287909
**55**	TRIM21	6737	tripartite motif containing 21	RNF81, RO52, Ro/SSA, SSA, SSA1	16539820
**56**	UROS	7390	uroporphyrinogen III synthase	UROIIIS	11021563

### GO functional analysis

**Figure 3:**
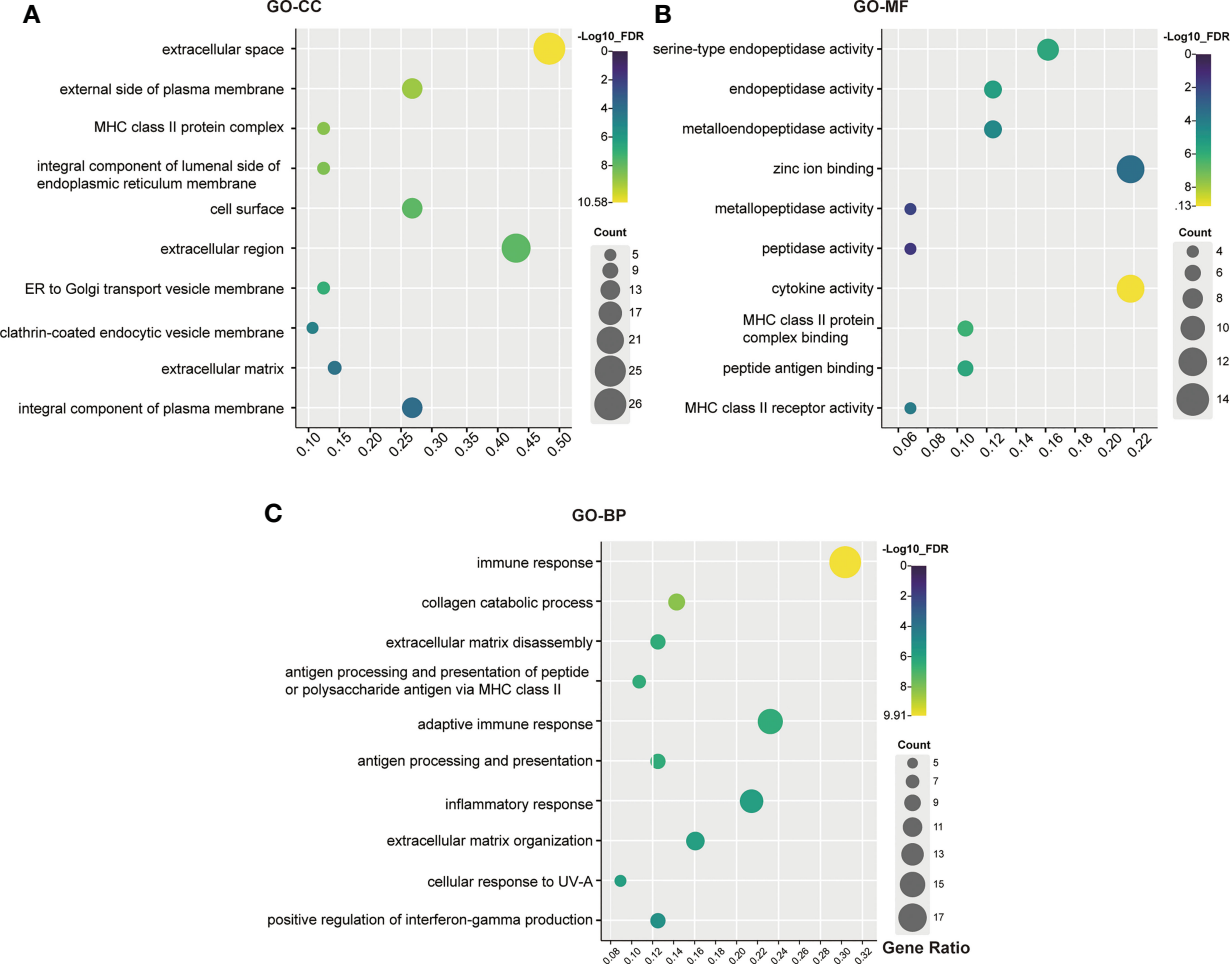
GO analysis bubble map of scleritis-associated genes. **(A)** Cellular components (CC); **(B)** Molecular functions (MF); **(C)** Biological process (BP). The circles highlighted in purple had higher FDRs, while those highlighted in yellow had lower FDRs. X-axis stands for the Gene Ratio of the pathway. Counts of pathways are represented by circles, and a larger circle indicates a pathway with a higher enrichment. (Crafting website: https://www.chiplot.online/).

To evaluate the biological characteristics of the identified genes and proteins, GO enrichment analysis was conducted using DAVID online tools. A total of 46 functions were included in the CC analysis, of which 24 were significantly enriched (FDR < 0.05). For the CC analysis, most of the genes and proteins identified were enriched in “extracellular space”, “external side of plasma membrane”, “MHC class II protein complex”, “integral component of lumenal side of endoplasmic reticulum membrane” and “cell surface”. In the MF analysis, 29 functions were involved, of which 17 were significantly enriched (FDR< 0.05). These genes were highly associated with “serine-type endopeptidase activity”, “endopeptidase activity”, “metalloendopeptidase activity”, “zinc ion binding” and “metallopeptidase activity”. The BP analysis identified 143 functions, 99 of which were significantly enriched (FDR < 0.05). The top 5 terms were “immune response”, “collagen catabolic process”, “extracellular matrix organization”, “antigen processing and presentation of peptide or polysaccharide antigen *via* MHC class II” and “antigen processing and presentation”. The top ten functional enrichment analyses for GO (CC, MF, BP) are shown in **Figure 3**. Furthermore, specific information on the significant GO terms is provided in **Supplementary Table S3**.

### KEGG analysis of genes and proteins

KEGG enrichment pathways were analyzed using DAVID online tools to show the enriched pathways associated with the identified candidate genes and proteins. As a result of KEGG analysis, 65 pathways were significantly altered (FDR<0.05) (**Supplementary Table S4**).

**Figure 4:**
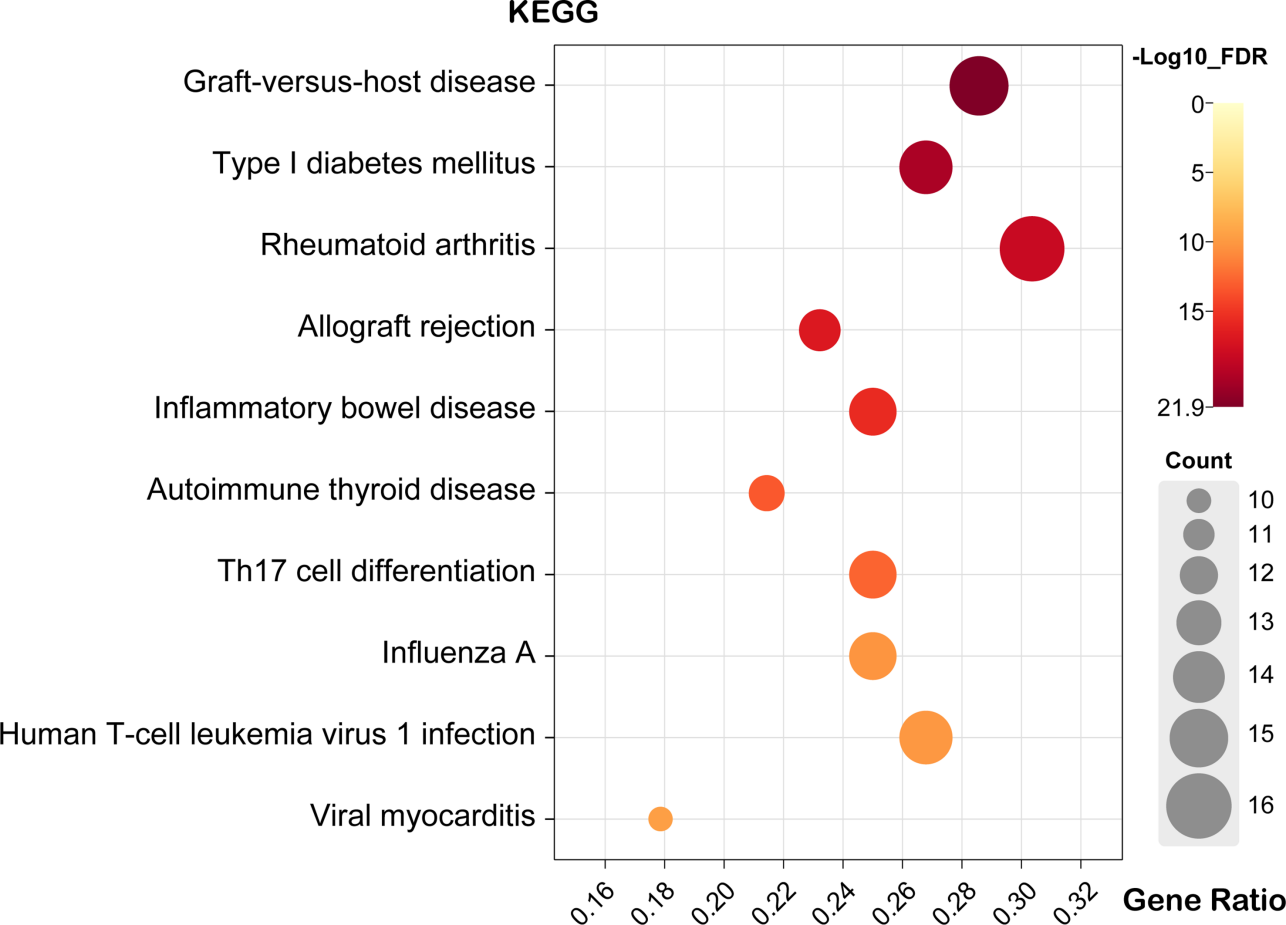
Bubble map of scleritis-associated genes for the top ten pathways of KEGG. X-axis stands for the Gene Ratio of the pathway. (Crafting website: https://www.chiplot.online/).

The top five pathways were “Graft-versus-host disease (GVHD)”, “Type I diabetes mellitus”, “RA”, “Allograft rejection” and “Inflammatory bowel disease (IBD)” (**Figure 4**). A detailed list of the corresponding genes enriched in KEGG analysis is provided in **Supplementary Table S4**. Moreover, the GVHD pathway was the most significant in the enrichment analysis (**Supplementary Figure S1.A**). The RA pathways had the largest number of genes, with a total of 17 genes enriched in these pathways (**Supplementary Figure S1.B**). Moreover, histocompatibility complex (MHC), TNF, IL1A, IL1β, IL2, IFNγ, and TNF were widely present in the top five disease signaling pathways (**Supplementary Table S4**), suggesting that these genes may be involved in the development of scleritis.

### PPI network construction and key module identification

**Figure 5:**
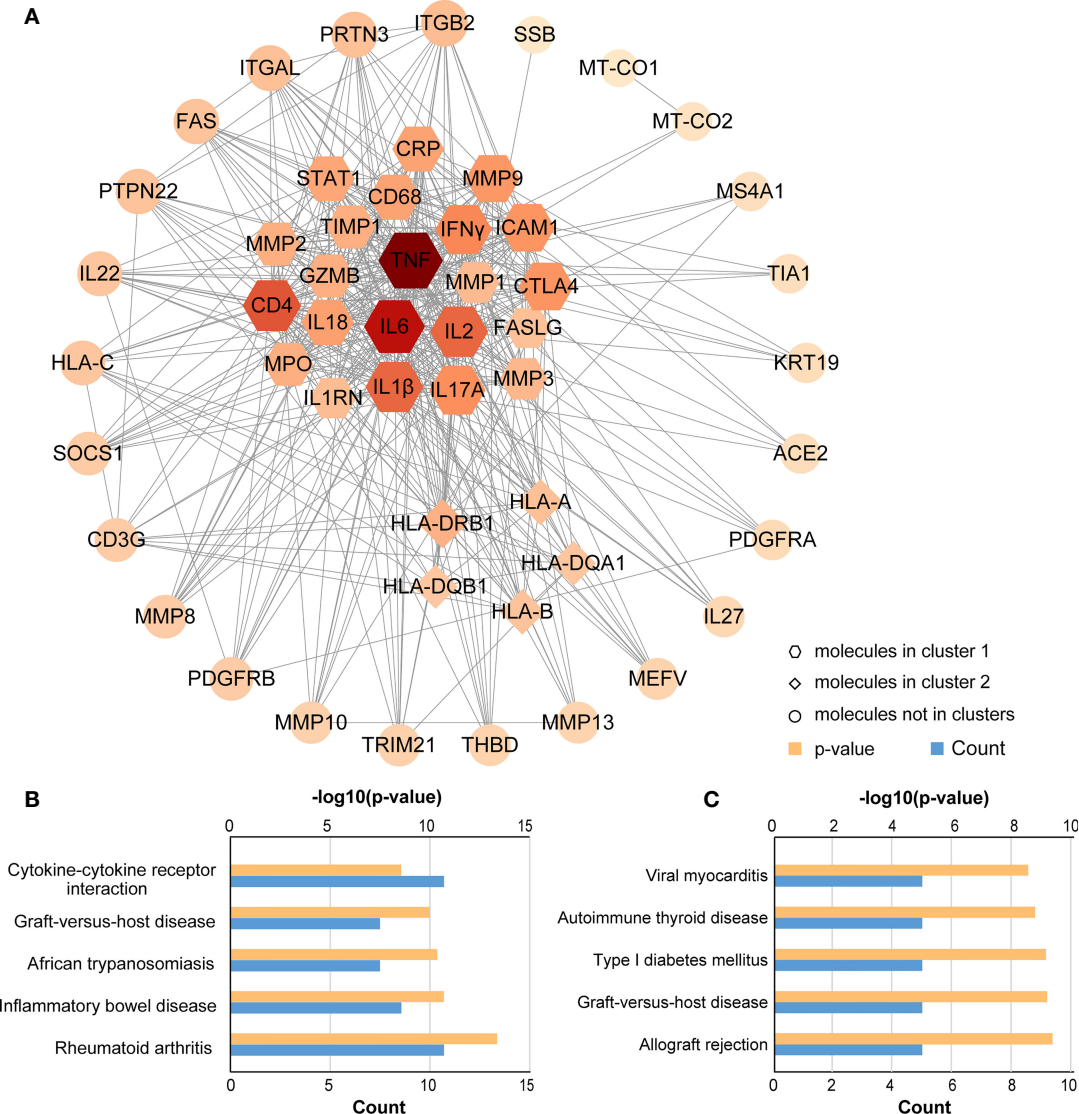
PPI network and KEGG pathway analysis for key clusters. **(A)** Construction and analysis of the PPI network. Hexagons represent cluster 1, diamonds represent cluster 2, and other circles showed interactions between multiple genes in the PPI network. The size and color of the graph are related to the degree. The bigger and more red, the higher degree on the map. **(B)** KEGG pathway analysis for cluster 1; **(C)** KEGG pathway analysis for cluster 2.

Using the STRING database, the PPI network was constructed and uploaded to Cytoscape for the purpose of searching key modules of genes and proteins. All 52 nodes and 896 edges of the PPI network are depicted in **Figure 5**. In addition, three main modules were identified with the MCODE plugin in Cytoscape. Cluster 1 (MCODE score = 18.857) consisted of 22 nodes and 198 edges, and cluster 2 (MCODE score = 5.000) had 5 nodes and 19 edges (**Figure 5**). Several genes were significantly enriched in cluster 1, including TNF, IL6, CD4, IL2, IFNγ, IL1β, ICAM1 and the MMP family of genes, most of which are related to intercellular signal transduction and protein hydrolysis. According to the KEGG pathway analysis of Cluster 1, these genes are involved in RA, IBD, African trypanosomiasis, GVHD and cytokine-cytokine receptor interaction. In cluster 2, the significantly enriched genes were HLA-B, HLA-A, HLA-DRB1, HLA-DQA1 and HLA-DQB1, with all genes related to allograft rejection, GVHD, type I diabetes, autoimmune thyroid disease and viral myocarditis.

### Identifying the hub genes

**Figure 6:**
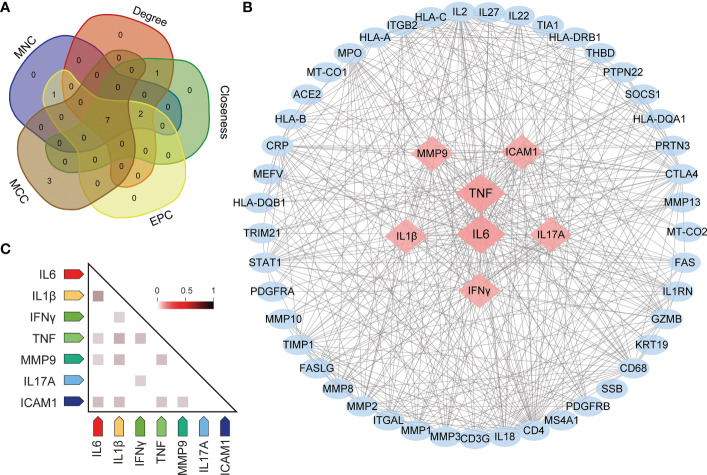
Screening for hub genes. **(A)** Hub genes are obtained in Venn analysis. **(B)** The scleritis-associated genes PPI network was constructed by Cytohubba with 7 hub genes and 45 other genes. Nodes in pink diamonds represent the top 7 hub genes, and other blue circles represent the other 45 genes, indicating gene interactions. **(C)** Coexpression analysis of seven hub genes in Homo sapiens.

To identify the most critical nodes in the PPI network, all nodes were ranked using five algorithms in CytoHubba: MNC, MCC, degree, EPC, and closeness. As shown in **Supplementary Table S5**, the top ten hub genes were identified with these five algorithms. The overlapping hub genes in the five algorithms were analyzed with VENN (**Figure 6A**). The overlapping genes based on VENN were identified as hub genes, namely, TNF, IL6, IFNγ, IL1β, ICAM1, IL17A and MMP9. Among all genes, TNF and IL6 had the highest combined scores (**Figure 6B**). Furthermore, gene coexpression analysis of the seven hub genes suggested that these hub genes might interact actively (**Figure 6C**).

### Validating the hub genes *in vitro*

**Figure 7:**
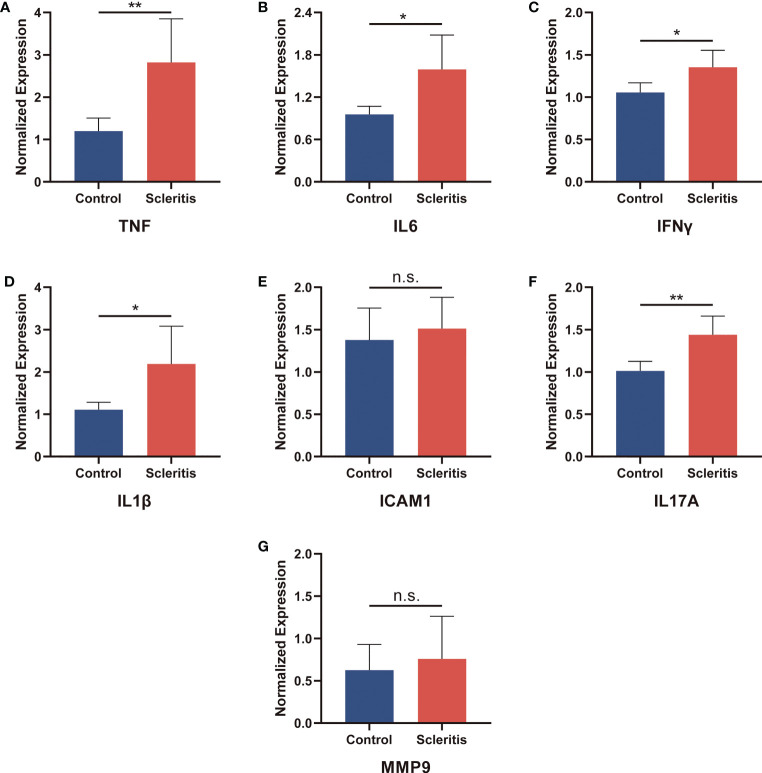
Validation of the relative protein expression levels in serum. **(A-G)** The levels of TNF, IL6, IFNγ, IL1β, ICAM1, IL17A and MMP9 in patients with active scleritis (n = 5) and healthy control subjects (n = 5). *P < 0.05; **P < 0.01; NS, no significant (two-tailed student’s t-test). Graphs show mean ± SEM.

To verify the protein expression levels of TNF, IL6, IFNγ, IL1β, ICAM1, IL17A and MMP9, serum samples from 5 healthy controls and 5 patients with active scleritis were collected to perform ELISA (**Figure 7**). The expressions of TNF, IL6, IFNγ, IL1β and IL17A in the serum of patients with active scleritis were significantly increased (*P* < 0.05). The expressions of ICAM1 and MMP9 were not significantly different between healthy controls and patients, but showed an increasing trend in patients. Additionally, we also investigated the expression of other important Inflammation-related cytokines such as IL4, IL12, TGFβ and HMGB1 (**Supplementary Figure S2**).

### Drug-gene interaction

**Figure 8:**
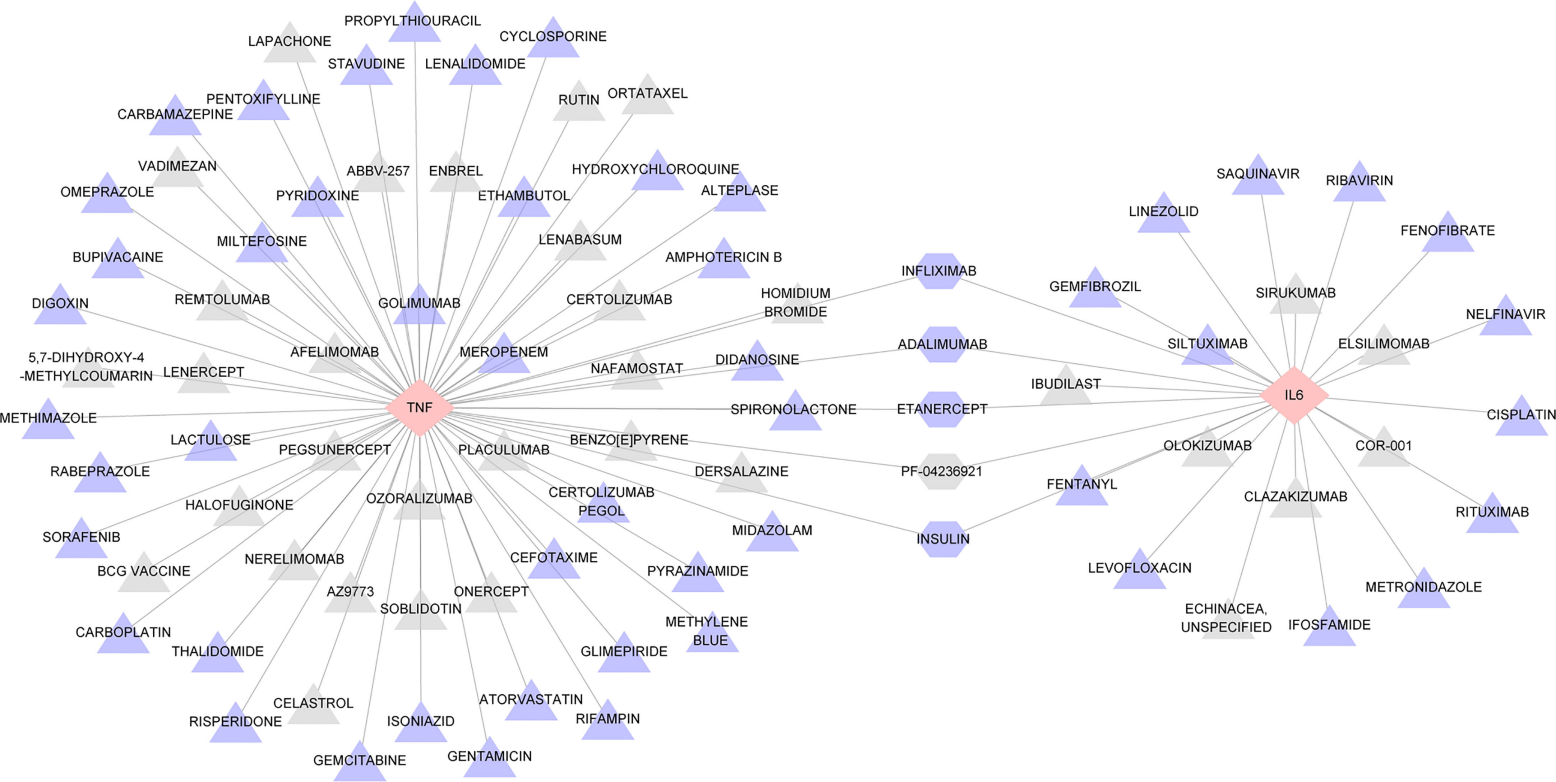
Interaction network between hub genes and targeted drugs. TNF and IL6, the two drug-targeted genes, are represented as pink diamonds. As identified therapeutic agents, 88 drugs were displayed in triangles and regularly arranged according to their score level; triangles closer to the center have higher scores. DALIMUMAB, ETANERCEPT, INFLIXIMAB, INSULIN, PF-04236921 represented by a hexagon. Drugs approved by FDA are shown in purple, while those not approved are shown in gray.

As key genes, TNF and IL6 were considered first-grade hub genes. We entered TNF and IL6 into the DGIdb to search for potential drugs. This analysis revealed 88 drugs, and **Supplementary Table S6** provides detailed information for these drugs, including the type of interaction, sources, PMIDs, interaction scores, and Food and Drug Administration (FDA) approval status. A higher score indicates greater importance. A review of the collected results shows that most drugs are antibodies and inhibitors. Among these illustrated drugs, ADALIMUMAB, ETANERCEPT, INFLIXIMAB, INSULIN, and PF-04236921 were predicted to target both TNF and IL6 (**Figure 8**).

### Identifying the hub genes and analyzing the drug-gene interaction according to different subtypes of scleritis

Since scleritis can be divided into different subtypes according to etiology or site of onset, clinical management strategies for them are different. Therefore, we further refined the collected candidate genes and proteins associated with scleritis (**Supplementary Table S2**). They were divided into infectious scleritis-associated and non-infectious scleritis-associated according to etiology, and anterior scleritis-associated and posterior scleritis-associated according to location. We performed the hub genes identification and drug-gene interaction analysis again as described previously. It was found that TNF was the highest-scoring hub gene in all scleritis subtypes, TNF and IL6 and their targeted drugs remained applicable to non-infectious scleritis and anterior scleritis. However, in addition to 68 drugs targeting TNF, four drugs targeting HLA-DQA1 were found to be potentially effective for infectious scleritis, nine drugs targeting CD4 were found to be potentially effective for posterior scleritis (**Supplementary Table S7** and **Figure S2**).

## Discussion

In the current study, we summarized and refined the genes and proteins associated with scleritis from the published literature. Through these candidate genes and proteins, we performed a systematic analysis to identify essential pathways and search for potential drugs. Two key modules and eight key genes were finally identified. Eighty-eight potential drugs were considered as candidates for the key genes TNF and IL6, and DALIMUMAB, ETANERCEPT, INFLIXIMAB, PF-04236921, and INSULIN were found to act against both TNF and IL6 and were identified as potentially contributing to the treatment of scleritis.

The results of GO concentration analysis showed that cytokines and MHC class II protein complexes have important roles in the development of inflammation in scleritis disease, as suggested in several reports (26–29). The top pathway enrichment analysis from the KEGG database was GVHD, which occurs in patients who have undergone allogeneic hematopoietic stem cell transplantation. It is caused by a series of “cytokine storms” stimulated after transplantation by T lymphocytes in the allogeneic donor graft that promote an immune response against the recipient’s antigens (30). Whereas ocular GVHD is predominantly characterized by inflammation of the ocular surface, cornea, conjunctiva, eyelids and lacrimal glands, less commonly, the posterior segment of the eye is involved in the form of microvascular retinopathy, scleritis or intraretinal and vitreous hemorrhage (31). Reports of GVHD-associated scleritis are rare, but we cannot ignore them in the clinic. Although ocular GVHD is not fatal, it has an extremely serious impact on a patient’s quality of life and ability to perform daily activities, so a more careful ocular evaluation for patients before transplantation, including pretransplant screening, will allow for early detection and treatment of ocular complications associated with GVHD and may prevent more dramatic outcomes (32). In addition, KEGG analysis showed the enrichment of many pathways associated with autoimmune diseases, such as type I diabetes, RA, IBD, autoimmune thyroid disease, leukemia and systemic lupus erythematosus (33–36). The relationship between type I diabetes and scleritis has received attention. There is limited information to accurately describe the relationship and clinical course of diabetes mellitus with scleritis. A few case reports suggest that diabetes may be a potential cause of infectious scleritis (37, 38). Studies that have attempted to characterize patients with scleritis have found that up to 20% of patients have an underlying diagnosis of diabetes mellitus (39), which raises red flags about the potential for catastrophic ocular disease in those with underlying diabetes mellitus. Furthermore, scleritis associated with RA has been widely reported. Scleritis is the most common ocular manifestation of RA, accounting for 25%-36% of all ocular involvement, while RA accounts for 8%-15% of scleritis cases (40). Treatment of scleritis in RA can be challenging and in severe cases can lead to loss of vision or even the eye.

In our study, two key genes, TNF and IL6, were eventually identified by CytoHubba and Venn analysis. TNF encodes a multifunctional proinflammatory cytokine belonging to the tumor necrosis factor superfamily. It can bind to and act through its receptors TNFRSF1A/TNFR1 and TNFRSF1B/TNFBR (41). TNF-α is the major active component of TNF, accounting for 70%-95% of the total, and several studies have shown that TNF-α participates in the pathogenesis of scleritis (42, 43). In addition, this cytokine has been associated with various diseases, including ankylosing spondylitis, tuberculosis, vasculitis, and many autoimmune diseases, some of which are closely related to scleritis (44–46). According to some studies, TNF-α is elevated in the tears and tissues of patients with necrotizing scleritis (47). Moreover, TNF-α has been shown to be significantly elevated in infiltrating inflammatory cells, especially plasma cells, compared to control cells and to induce an increase in matrix metalloproteinase content (48). On the other hand, we found that IL6 plays an important role in scleritis. It encodes a cytokine that has an important effect on inflammation and B-cell maturation (49). IL6 can be produced by fibroblasts, monocytes/macrophages, B cells, T cells, and various cancer cells. IL6 is involved in the growth, differentiation and functional regulation of a variety of cells and plays an important role in systematic immune and inflammatory responses (50). In an *in vitro* experiment, scleral cells stimulated with IL1β showed a significant increase in the expression level of IL6 compared to control cells (51), indicating that IL6 is involved in an important reaction process in the inflammation of the sclera. In addition, in patients with scleritis associated with giant cell arteritis, anti-IL6 drugs have been tested in those with several relapses, corticosteroid dependence and resistance to methotrexate (52). These results support our findings that TNF and IL6 are key mediators associated with scleritis, suggesting that they may be attractive drug targets for the treatment of scleritis.

We made network diagrams by drug-gene interactions to find targeted drugs that can interact with TNF and IL6. From the results obtained with DGIdb, most of the 88 targeted drugs were inhibitors, monoclonal antibodies, etc. According to the results of the analysis, a total of five drugs were specific for both TNF and IL6, namely, INFLIXIMAB, ADALIMUMAB, ETANERCEPT, PF-04236921, and INSULIN, among which PF-04236921 has not been currently recognized by the FDA (40, 53, 54). As previously reported, INFLIXIMAB and ADALIMUMAB have achieved satisfactory clinical efficacy in the treatment of ocular inflammatory and systemic autoimmune diseases (55, 56). The current research on the mechanism of action of these two biological agents mainly focuses on targeting TNF-α. However, according to the results of DGIdb database analysis, IL6 is also a potential target of infliximab and adalimumab, and there is no related research so far, and more basic research and clinical trials are needed in the future. Since ETANERCEPT’s first introduction in 1998, it has shown a significantly beneficial effect on the treatment of RA. ETANERCEPT has also been increasingly used in the treatment of psoriatic arthritis and ankylosing spondylitis, with favorable results in randomized controlled trials, but it has been reported to have adverse effects, including inflammatory ophthalmopathy (57). Current studies are limited, and it is challenging to establish a true link between ETANERCEPT and inflammation (58). Since ETANERCEPT is an anti-TNF drug with a different mechanism of action, it has been hypothesized that the imbalance between TNF-α and its soluble and membrane-bound cell receptor levels leads to the paradoxical performance of the treatment (59). Therefore, it is necessary to be cautious about whether to choose this drug, and more exploration and research are needed on how to apply it in clinical practice. PF-04236921 is a fully humanized IgG2 monoclonal antibody now at the stages of clinical testing in autoimmune diseases (60, 61), possessing important reference significance and application prospects for scleritis related to autoimmune diseases. INSULIN is a protein hormone secreted by islet β-cells in the pancreas that is stimulated by endogenous or exogenous substances. Published literature indicates that the role of tumor necrosis factor in the development of insulin resistance is well established, but the mechanism of interaction in humans has not yet been reported. In the case of IL6, insulin resistance and stimulation of IL6 seem to be mechanistically related (62). Currently, an increasing number of basic experiments and clinical studies have demonstrated that the modulation of insulin signaling has a considerable role in treating neurodegenerative disease (63–65). Insulin has anti-inflammatory effects, and the application of insulin can reduce pro-inflammatory mediators, especially TNFα and IL6, in animal models of endotoxemia (66–68). Although no information has been reported on insulin for the treatment of scleritis, the role of insulin in treating inflammation cannot be underestimated. Intensive insulin therapy with close, continuous monitoring of plasma glucose levels is feasible in clinical practice in major hospitals (69). Glucose-insulin-potassium (GIK) regimen in patients with ischemic heart disease (acute myocardial infarction) or IBD has been found to inhibit inflammatory processes and appear to be cytoprotective (70–72). Therefore, establishing a link between insulin and scleritis is prospective, and further studies are needed to determine the effectiveness of insulin in treating scleritis.

Since scleritis can be divided into different subtypes according to the etiology or location of disease, the clinical treatment strategies are also different, we further refine the analysis results to identify the potential hub genes and targeted drugs of different subtypes of scleritis. The results confirmed that our prediction of scleritis hub genes and targeted drugs is more suitable for non-infectious scleritis and anterior scleritis, but for infectious scleritis and posterior scleritis, the current analysis methods cannot accurately locate the target drug. This may be due to the small number of cases and the unclear description of the subtype of scleritis in the reports, which requires further study.

Some limitations exist in the present study. First, from the data sources, the data analyzed in our study are from the published literature, which has restricted the total amount and scope of data. New studies may have an unpredictable impact on the current results. Second, we could not perform bioinformatic network analysis or gene expression prediction due to a lack of epigenetic data. Third, some bioactive lipids may also play an important role in the pathogenesis of scleritis (73), but due to the current limited number of case reports and detection indicators, further exploration is needed. Finally, our research focuses on two important genes, TNF and IL6, and drug candidates have been postulated based on them. Currently, INFLIXIMAB and ADALIMUMAB have been used in the clinical treatment of scleritis. In refractory scleritis, experts may consider alternative or combination drugs to relieve patients’ symptoms. However, the effect of ETANERCEPT is still controversial, and PF-04236921 is currently still being tested in clinical trials and may become a promising drug. The association of INSULIN with scleritis is still in the predictive stage, and further research is necessary. This study provides comprehensive bioinformatics information on target genes and drug candidates for the treatment of scleritis. It may be possible to gain a deeper understanding of the molecular mechanisms involved in this disease based on this information. Nevertheless, biomarkers and drugs need to be investigated further to determine their mechanisms and specific functions in disease pathogenesis.

## Data availability statement

The original contributions presented in the study are included in the article/**Supplementary Material**. Further inquiries can be directed to the corresponding author.

## Ethics statement

The studies involving human participants were reviewed and approved by the Research Ethics Committee of the Renmin Hospital of Wuhan University. Written informed consent to participate in this study was provided by the participants’ legal guardian/next of kin.

## Author contributions

FY: Conceptualization, Methodology, Formal Analysis, Writing - Original Draft; YL: Data Curation, Writing - Original Draft; TZ: Validation, Review & Editing; YS: Conceptualization, Funding Acquisition, Resources, Supervision, Writing - Review & Editing. All authors contributed to the article and approved the submitted version.
