# Supplementary material for: Identifying TNF and IL6 as potential hub genes and targeted drugs associated with scleritis: A bio-informative report
Source: Front Immunol. 2023 Mar 31;14:1098140. doi: 10.3389/fimmu.2023.1098140 (PMC10102337; doi:10.3389/fimmu.2023.1098140)
Supplement: Supplementary file 1 [file Table_1.docx]

**Supplementary Table S1.** Summary of the clinical information of the participants.

| **Participant** | **Age** | **Medication use at the time of sample collection** | **Diagnosis** |
| --- | --- | --- | --- |
| 1 | 40-50 | Prednisone | Scleritis |
| 2 | 30-40 | Unmedicated | Scleritis |
| 3 | 30-40 | TobraDex | Scleritis |
| 4 | 10-20 | Unmedicated | Scleritis |
| 5 | 40-50 | Ibuprofen | Scleritis |
| 6 | 20-30 | n/a | Healthy control |
| 7 | 30-40 | n/a | Healthy control |
| 8 | 20-30 | n/a | Healthy control |
| 9 | 30-40 | n/a | Healthy control |
| 10 | 40-50 | n/a | Healthy control |
