# Supplementary material for: Identifying TNF and IL6 as potential hub genes and targeted drugs associated with scleritis: A bio-informative report
Source: Front Immunol. 2023 Mar 31;14:1098140. doi: 10.3389/fimmu.2023.1098140 (PMC10102337; doi:10.3389/fimmu.2023.1098140)
Supplement: Supplementary file 2 [file Table_2.docx]

**Supplementary Table S2** Scleritis-associated genes with data sources, regulation of pathogenesis, and classification by etiology and location.

| **Number** | **Gene**  **Symbol** | **Data sources** | **Regulation of the pathogenesis** | **Etiology** | **Location of disease** |  |  |
| --- | --- | --- | --- | --- | --- | --- | --- |
| 1 | ACE2 | ocular tissues | Negative | Infectious | anterior |  | |
| 2 | CD3G | ocular tissues | Negative | non-infection | anterior |  |  |
| 3 | CD4 | ocular tissues | Negative | non-infection | posterior |  |  |
| 4 | CD68 | ocular tissues | Positive | non-infection | anterior |  |  |
| 5 | CRP | non-ocular tissues | Positive | non-infection | posterior |  |  |
| 6 | CTLA4 | non-ocular tissues | Positive | non-infection | anterior and posterior |  |  |
| 7 | FAS | ocular tissues | Positive | non-infection | anterior |  |  |
| 8 | FASLG | ocular tissues | Positive | non-infection | anterior |  |  |
| 9 | FCGBP | non-ocular tissues | Positive | non-infection | anterior |  |  |
| 10 | GZMB | tears | Positive | non-infection | anterior |  |  |
| 11 | HLA-A | non-ocular tissues | Positive | non-infection | posterior |  |  |
| 12 | HLA-B | non-ocular tissues | Positive | non-infection | anterior and posterior |  |  |
| 13 | HLA-C | non-ocular tissues | Positive | Infectious | n/a |  |  |
| 14 | HLA-DQA1 | non-ocular tissues | Negative | Infectious | n/a |  |  |
| 15 | HLA-DQB1 | non-ocular tissues | Negative | Infectious | n/a |  |  |
| 16 | HLA-DRB1 | non-ocular tissues | Negative | Infectious | n/a |  |  |
| 17 | HLA-DRB4 | non-ocular tissues | Negative | Infectious | n/a |  |  |
| 18 | ICAM1 | ocular tissues | Positive | non-infection | anterior |  |  |
| 19 | IFNγ | non-ocular tissues | Negative | n/a | n/a |  |  |
| 20 | IGAN1 | non-ocular tissues | n/a | non-infection | anterior |  |  |
| 21 | IL17 | non-ocular tissues | Positive | n/a | n/a |  |  |
| 22 | IL18 | ocular tissues | Positive | non-infection | n/a |  |  |
| 23 | IL1β | ocular tissues | Positive | non-infection | n/a |  |  |
| 24 | IL1RA | ocular tissues | Negative | non-infection | anterior |  |  |
| 25 | IL2 | non-ocular tissues | Positive | n/a | n/a |  |  |
| 26 | IL22 | non-ocular tissues | Positive | non-infection | anterior |  |  |
| 27 | IL27 | non-ocular tissues | Negative | n/a | n/a |  |  |
| 28 | IL6 | non-ocular tissues | Positive | non-infection | anterior and posterior |  |  |
| 29 | ITGAL | ocular tissues | Positive | n/a | anterior |  |  |
| 30 | ITGB2 | ocular tissues | Positive | non-infection | anterior |  |  |
| 31 | KRT19 | ocular tissues | Positive | non-infection | anterior |  |  |
| 32 | MEFV | ocular tissues | n/a | non-infection | posterior |  |  |
| 33 | MMP1 | ocular tissues | Positive | non-infection | n/a |  |  |
| 34 | MMP10 | on-ocular tissues | Positive | non-infection | n/a |  |  |
| 35 | MMP13 | on-ocular tissues | Positive | non-infection | n/a |  |  |
| 36 | MMP2 | non-ocular tissues | Positive | non-infection | n/a |  |  |
| 37 | MMP3 | ocular tissues | Positive | non-infection | n/a |  |  |
| 38 | MMP8 | ocular tissues | Positive | non-infection | n/a |  |  |
| 39 | MMP9 | ocular tissues | Positive | non-infection | n/a |  |  |
| 40 | MPO | non-ocular tissues | Positive | non-infection | anterior |  |  |
| 41 | MS4A1 | non-ocular tissues | Positive | non-infection | anterior |  |  |
| 42 | MT-CO1 | non-ocular tissues | Negative | non-infection | n/a |  |  |
| 43 | MT-CO2 | non-ocular tissues | Negative | n/a | anterior |  |  |
| 44 | PDGFRA | ocular tissues | Positive | non-infection | n/a |  |  |
| 45 | PDGFRB | ocular tissues | Positive | non-infection | n/a |  |  |
| 46 | PRTN3 | non-ocular tissues | Positive | non-infection | anterior and posterior |  |  |
| 47 | PTPN22 | non-ocular tissues | Positive | non-infection | anterior and posterior |  |  |
| 48 | SOCS1 | non-ocular tissues | Negative | non-infection | n/a |  |  |
| 49 | SSB | non-ocular tissues | Positive | non-infection | posterior |  |  |
| 50 | STAT1 | non-ocular tissues | n/a | n/a | n/a |  |  |
| 51 | THBD | ocular tissues | Positive | non-infection | n/a |  |  |
| 52 | TIA1 | ocular tissues | Positive | non-infection | anterior |  |  |
| 53 | TIMP1 | ocular tissues | Negative | non-infection | n/a |  |  |
| 54 | TNF | non-ocular tissues | Positive | infection and non-infection | anterior and posterior |  |  |
| 55 | TRIM21 | non-ocular tissues | Positive | non-infection | n/a |  |  |
| 56 | UROS | non-ocular tissues | n/a | non-infection | n/a |  |  |
