# Supplementary material for: Identifying TNF and IL6 as potential hub genes and targeted drugs associated with scleritis: A bio-informative report
Source: Front Immunol. 2023 Mar 31;14:1098140. doi: 10.3389/fimmu.2023.1098140 (PMC10102337; doi:10.3389/fimmu.2023.1098140)
Supplement: Supplementary file 3 [file Table_3.docx]

**Supplementary Table S3** All functional enrichment analyses of gene ontology (GO)

| Number | classification | Term | Genes | Count | Gene Ratio (%) | FDR |
| --- | --- | --- | --- | --- | --- | --- |
| 1 | BP | GO:0006955~immune response | HLA-DRB4, HLA-B, IL18, HLA-C, FASLG, HLA-A, TNF, IL2, IL1A, CD4, IL1B, FAS, CTLA4, HLA-DQA1, HLA-DRB1, HLA-DQB1, IL17A | 17 | 30.3571429 | 1.23E-10 |
| 2 | BP | GO:0030574~collagen catabolic process | MMP13, MMP1, MMP2, MMP3, PRTN3, MMP8, MMP9, MMP10 | 8 | 14.2857143 | 6.54E-09 |
| 3 | BP | GO:0022617~extracellular matrix disassembly | MMP13, MMP1, MMP2, MMP3, MMP8, MMP9, MMP10 | 7 | 12.5 | 4.22E-07 |
| 4 | BP | GO:0002504~antigen processing and presentation of peptide or polysaccharide antigen via MHC class II | HLA-DRB4, HLA-B, HLA-A, HLA-DRB1, HLA-DQA1, HLA-DQB1 | 6 | 10.7142857 | 4.22E-07 |
| 5 | BP | GO:0002250~adaptive immune response | HLA-DRB4, HLA-B, HLA-C, CD3G, HLA-A, IL2, CD4, IFNG, CTLA4, HLA-DQA1, HLA-DRB1, HLA-DQB1, IL17A | 13 | 23.2142857 | 4.22E-07 |
| 6 | BP | GO:0019882~antigen processing and presentation | HLA-DRB4, HLA-B, HLA-C, HLA-A, HLA-DRB1, HLA-DQA1, HLA-DQB1 | 7 | 12.5 | 4.22E-07 |
| 7 | BP | GO:0006954~inflammatory response | CRP, IL1A, IL22, IL6, IL1B, ITGB2, IL18, IL27, MEFV, ITGAL, TNF, IL17A | 12 | 21.4285714 | 1.46E-06 |
| 8 | BP | GO:0030198~extracellular matrix organization | PDGFRA, MMP13, MMP1, MMP2, MMP3, MMP8, TNF, MMP9, MMP10 | 9 | 16.0714286 | 1.48E-06 |
| 9 | BP | GO:0071492~cellular response to UV-A | MMP1, MMP2, MMP3, TIMP1, MMP9 | 5 | 8.92857143 | 1.48E-06 |
| 10 | BP | GO:0032729~positive regulation of interferon-gamma production | IL1B, IL18, IL27, PTPN22, HLA-A, TNF, IL2 | 7 | 12.5 | 6.13E-06 |
| 11 | BP | GO:0045429~positive regulation of nitric oxide biosynthetic process | IFNG, IL1B, ITGB2, MMP8, TNF, ICAM1 | 6 | 10.7142857 | 1.64E-05 |
| 12 | BP | GO:0045429~positive regulation of nitric oxide biosynthetic process | IFNG, IL1B, ITGB2, MMP8, TNF, ICAM1 | 6 | 10.7142857 | 1.64E-05 |
| 13 | BP | GO:0032755~positive regulation of interleukin-6 production | IL1A, IL6, IFNG, IL1B, MMP8, TNF, IL17A | 7 | 12.5 | 2.65E-05 |
| 14 | BP | GO:0032755~positive regulation of interleukin-6 production | IL1A, IL6, IFNG, IL1B, MMP8, TNF, IL17A | 7 | 12.5 | 2.65E-05 |
| 15 | BP | GO:0030225~macrophage differentiation | CD4, SOCS1, IFNG, MMP9, HLA-DRB1 | 5 | 8.92857143 | 3.81E-05 |
| 16 | BP | GO:0006959~humoral immune response | IL6, IFNG, MS4A1, TNF, HLA-DRB1, HLA-DQB1 | 6 | 10.7142857 | 3.91E-05 |
| 17 | BP | GO:0050830~defense response to Gram-positive bacterium | CRP, IL6, IL1B, IL18, HLA-A, TNF, IL17A | 7 | 12.5 | 7.07E-05 |
| 18 | BP | GO:0048661~positive regulation of smooth muscle cell proliferation | PDGFRB, IL6, STAT1, MMP2, IL18, TNF | 6 | 10.7142857 | 7.07E-05 |
| 19 | BP | GO:0050830~defense response to Gram-positive bacterium | CRP, IL6, IL1B, IL18, HLA-A, TNF, IL17A | 7 | 12.5 | 7.07E-05 |
| 20 | BP | GO:0006508~proteolysis | THBD, ACE2, MMP13, MMP1, MMP2, MMP3, PRTN3, MMP8, MMP9, MMP10 | 10 | 17.8571429 | 8.63E-05 |
| 21 | BP | GO:0050870~positive regulation of T cell activation | CD4, HLA-DRB4, HLA-DRB1, HLA-DQA1, HLA-DQB1 | 5 | 8.92857143 | 8.75E-05 |
| 22 | BP | GO:0070374~positive regulation of ERK1 and ERK2 cascade | PDGFRB, IL1A, PDGFRA, CD4, PTPN22, TNF, HLA-DRB1, ICAM1 | 8 | 14.2857143 | 1.24E-04 |
| 23 | BP | GO:1904996~positive regulation of leukocyte adhesion to vascular endothelial cell | IL6, ITGB2, TNF, ICAM1 | 4 | 7.14285714 | 2.81E-04 |
| 24 | BP | GO:1904645~response to beta-amyloid | MMP13, MMP2, MMP3, MMP9 | 4 | 7.14285714 | 3.34E-04 |
| 25 | BP | GO:0002503~peptide antigen assembly with MHC class II protein complex | HLA-DRB4, HLA-DRB1, HLA-DQA1, HLA-DQB1 | 4 | 7.14285714 | 3.92E-04 |
| 26 | BP | GO:0002381~immunoglobulin production involved in immunoglobulin mediated immune response | HLA-DRB4, HLA-DRB1, HLA-DQA1, HLA-DQB1 | 4 | 7.14285714 | 4.31E-04 |
| 27 | BP | GO:0050729~positive regulation of inflammatory response | IFNG, IL1B, IL18, MEFV, TNF, IL2 | 6 | 10.7142857 | 4.31E-04 |
| 28 | BP | GO:0050729~positive regulation of inflammatory response | IFNG, IL1B, IL18, MEFV, TNF, IL2 | 6 | 10.7142857 | 4.31E-04 |
| 29 | BP | GO:0043123~positive regulation of I-kappaB kinase/NF-kappaB signaling | IL1A, CD4, IL1B, FASLG, TRIM21, TNF, HLA-DRB1 | 7 | 12.5 | 4.31E-04 |
| 30 | BP | GO:0050852~T cell receptor signaling pathway | CTLA4, CD3G, PTPN22, HLA-A, HLA-DRB1, HLA-DQB1 | 6 | 10.7142857 | 4.31E-04 |
| 31 | BP | GO:0007568~aging | PDGFRB, MT-CO1, MMP2, ITGB2, TIMP1, CD68, MPO | 7 | 12.5 | 4.53E-04 |
| 32 | BP | GO:0060559~positive regulation of calcidiol 1-monooxygenase activity | IFNG, IL1B, TNF | 3 | 5.35714286 | 6.40E-04 |
| 33 | BP | GO:0001934~positive regulation of protein phosphorylation | CD4, IFNG, IL1B, FAS, TNF, MMP9, HLA-DRB1 | 7 | 12.5 | 6.77E-04 |
| 34 | BP | GO:0032731~positive regulation of interleukin-1 beta production | IL6, IFNG, MEFV, TNF, IL17A | 5 | 8.92857143 | 8.14E-04 |
| 35 | BP | GO:0032731~positive regulation of interleukin-1 beta production | IL6, IFNG, MEFV, TNF, IL17A | 5 | 8.92857143 | 8.14E-04 |
| 36 | BP | GO:0043065~positive regulation of apoptotic process | PDGFRB, IL6, MMP2, FAS, CTLA4, FASLG, TNF, MMP9 | 8 | 14.2857143 | 8.46E-04 |
| 37 | BP | GO:0006915~apoptotic process | IL1A, TIA1, IFNG, IL1B, ITGB2, FAS, GZMB, FASLG, MMP9, IL17A | 10 | 17.8571429 | 9.13E-04 |
| 38 | BP | GO:0042531~positive regulation of tyrosine phosphorylation of STAT protein | IL6, IFNG, IL18, TNF, IL2 | 5 | 8.92857143 | 9.37E-04 |
| 39 | BP | GO:0042531~positive regulation of tyrosine phosphorylation of STAT protein | IL6, IFNG, IL18, TNF, IL2 | 5 | 8.92857143 | 9.37E-04 |
| 40 | BP | GO:0033674~positive regulation of kinase activity | PDGFRB, PDGFRA, CD4, HLA-DRB1, IL2 | 5 | 8.92857143 | 0.00113594 |
| 41 | BP | GO:0045840~positive regulation of mitotic nuclear division | PDGFRB, IL1A, IL1B, TNF | 4 | 7.14285714 | 0.00173682 |
| 42 | BP | GO:0051092~positive regulation of NF-kappaB transcription factor activity | IL1B, ITGB2, IL18, TRIM21, TNF, ICAM1 | 6 | 10.7142857 | 0.001762 |
| 43 | BP | GO:0051092~positive regulation of NF-kappaB transcription factor activity | IL1B, ITGB2, IL18, TRIM21, TNF, ICAM1 | 6 | 10.7142857 | 0.001762 |
| 44 | BP | GO:0019886~antigen processing and presentation of exogenous peptide antigen via MHC class II | HLA-DRB4, HLA-DRB1, HLA-DQA1, HLA-DQB1 | 4 | 7.14285714 | 0.00176767 |
| 45 | BP | GO:0035987~endodermal cell differentiation | MMP2, ITGB2, MMP8, MMP9 | 4 | 7.14285714 | 0.00176767 |
| 46 | BP | GO:0097192~extrinsic apoptotic signaling pathway in absence of ligand | IL1A, IL1B, FAS, IL2 | 4 | 7.14285714 | 0.00246382 |
| 47 | BP | GO:0008284~positive regulation of cell proliferation | PDGFRB, PDGFRA, IL6, IFNG, IL1B, FASLG, PRTN3, TIMP1, IL2 | 9 | 16.0714286 | 0.00246382 |
| 48 | BP | GO:0010573~vascular endothelial growth factor production | IL6, IL1B, TNF | 3 | 5.35714286 | 0.00301741 |
| 49 | BP | GO:0071346~cellular response to interferon-gamma | STAT1, FASLG, TNF, ICAM1, HLA-DQB1 | 5 | 8.92857143 | 0.00301741 |
| 50 | BP | GO:0031663~lipopolysaccharide-mediated signaling pathway | IL1B, IL18, PTPN22, TNF | 4 | 7.14285714 | 0.00312721 |
| 51 | BP | GO:0071222~cellular response to lipopolysaccharide | IL1A, IL6, IL1B, CD68, TNF, ICAM1 | 6 | 10.7142857 | 0.00337568 |
| 52 | BP | GO:0071222~cellular response to lipopolysaccharide | IL1A, IL6, IL1B, CD68, TNF, ICAM1 | 6 | 10.7142857 | 0.00337568 |
| 53 | BP | GO:0097527~necroptotic signaling pathway | FAS, FASLG, TNF | 3 | 5.35714286 | 0.00360196 |
| 54 | BP | GO:0002486~antigen processing and presentation of endogenous peptide antigen via MHC class I via ER pathway, TAP-independent | HLA-B, HLA-C, HLA-A | 3 | 5.35714286 | 0.00360196 |
| 55 | BP | GO:0032760~positive regulation of tumor necrosis factor production | IL1A, IL6, IFNG, MMP8, IL17A | 5 | 8.92857143 | 0.0037695 |
| 56 | BP | GO:0032760~positive regulation of tumor necrosis factor production | IL1A, IL6, IFNG, MMP8, IL17A | 5 | 8.92857143 | 0.0037695 |
| 57 | BP | GO:0032722~positive regulation of chemokine production | IL6, IFNG, IL18, TNF | 4 | 7.14285714 | 0.00429957 |
| 58 | BP | GO:0050796~regulation of insulin secretion | IL6, IFNG, IL1B, TNF | 4 | 7.14285714 | 0.0047943 |
| 59 | BP | GO:0050796~regulation of insulin secretion | IL6, IFNG, IL1B, TNF | 4 | 7.14285714 | 0.0047943 |
| 60 | BP | GO:0010888~negative regulation of lipid storage | CRP, IL6, TNF | 3 | 5.35714286 | 0.00530706 |
| 61 | BP | GO:0097191~extrinsic apoptotic signaling pathway | IFNG, FAS, FASLG, TNF | 4 | 7.14285714 | 0.00621766 |
| 62 | BP | GO:0007267~cell-cell signaling | IL1B, ITGB2, IL18, FASLG, IL2, IL17A | 6 | 10.7142857 | 0.00641467 |
| 63 | BP | GO:0071407~cellular response to organic cyclic compound | STAT1, IL1B, IL18, TNF | 4 | 7.14285714 | 0.00693064 |
| 64 | BP | GO:0031334~positive regulation of protein complex assembly | IFNG, MMP1, MMP3, TNF | 4 | 7.14285714 | 0.00693064 |
| 65 | BP | GO:0016045~detection of bacterium | HLA-B, HLA-A, HLA-DRB1 | 3 | 5.35714286 | 0.00705125 |
| 66 | BP | GO:0010628~positive regulation of gene expression | CRP, IL1A, IL6, IFNG, IL1B, PTPN22, MMP8, TNF | 8 | 14.2857143 | 0.00791749 |
| 67 | BP | GO:0010628~positive regulation of gene expression | CRP, IL1A, IL6, IFNG, IL1B, PTPN22, MMP8, TNF | 8 | 14.2857143 | 0.00791749 |
| 68 | BP | GO:0071230~cellular response to amino acid stimulus | PDGFRA, SOCS1, MMP2, TNF | 4 | 7.14285714 | 0.00807343 |
| 69 | BP | GO:0046598~positive regulation of viral entry into host cell | CD4, TRIM21, HLA-DRB1 | 3 | 5.35714286 | 0.01058175 |
| 70 | BP | GO:0046688~response to copper ion | IL1A, MT-CO1, ICAM1 | 3 | 5.35714286 | 0.01186759 |
| 71 | BP | GO:0019221~cytokine-mediated signaling pathway | IL1A, IL6, SOCS1, STAT1, IL1B | 5 | 8.92857143 | 0.01187188 |
| 72 | BP | GO:0044267~cellular protein metabolic process | MMP13, MMP1, MMP2 | 3 | 5.35714286 | 0.01218699 |
| 73 | BP | GO:1901224~positive regulation of NIK/NF-kappaB signaling | IL1B, IL18, MMP8, TNF | 4 | 7.14285714 | 0.01218699 |
| 74 | BP | GO:0048143~astrocyte activation | IFNG, IL1B, TNF | 3 | 5.35714286 | 0.01218699 |
| 75 | BP | GO:0032496~response to lipopolysaccharide | THBD, IL1B, FASLG, PTPN22, MPO | 5 | 8.92857143 | 0.01218699 |
| 76 | BP | GO:0043410~positive regulation of MAPK cascade | IL6, CD4, MMP8, TNF, HLA-DRB1 | 5 | 8.92857143 | 0.01218699 |
| 77 | BP | GO:0051044~positive regulation of membrane protein ectodomain proteolysis | IFNG, IL1B, TNF | 3 | 5.35714286 | 0.01347894 |
| 78 | BP | GO:0045087~innate immune response | CRP, HLA-B, HLA-C, IL27, HLA-A, MEFV, TRIM21, IL17A | 8 | 14.2857143 | 0.01518577 |
| 79 | BP | GO:0014068~positive regulation of phosphatidylinositol 3-kinase signaling | PDGFRB, PDGFRA, IL18, TNF | 4 | 7.14285714 | 0.01826123 |
| 80 | BP | GO:0060252~positive regulation of glial cell proliferation | IL6, IL1B, TNF | 3 | 5.35714286 | 0.02113342 |
| 81 | BP | GO:0045672~positive regulation of osteoclast differentiation | IFNG, TNF, IL17A | 3 | 5.35714286 | 0.02236596 |
| 82 | BP | GO:0050768~negative regulation of neurogenesis | IL6, IL1B, TNF | 3 | 5.35714286 | 0.02236596 |
| 83 | BP | GO:0007165~signal transduction | PDGFRB, IL22, CD4, STAT1, IL1B, FAS, FASLG, IL27, TIMP1, ITGAL, HLA-DRB1 | 11 | 19.6428571 | 0.02342371 |
| 84 | BP | GO:0032740~positive regulation of interleukin-17 production | IL6, IL18, IL2 | 3 | 5.35714286 | 0.02552551 |
| 85 | BP | GO:0001916~positive regulation of T cell mediated cytotoxicity | HLA-B, HLA-A, HLA-DRB1 | 3 | 5.35714286 | 0.02643299 |
| 86 | BP | GO:0046330~positive regulation of JNK cascade | IL1A, IL1B, MMP8, TNF | 4 | 7.14285714 | 0.02643299 |
| 87 | BP | GO:0030101~natural killer cell activation | ITGB2, IL18, IL2 | 3 | 5.35714286 | 0.02643299 |
| 88 | BP | GO:0034341~response to interferon-gamma | STAT1, MEFV, TRIM21 | 3 | 5.35714286 | 0.02804742 |
| 89 | BP | GO:1900017~positive regulation of cytokine production involved in inflammatory response | IL6, TNF, IL17A | 3 | 5.35714286 | 0.02968673 |
| 90 | BP | GO:0010575~positive regulation of vascular endothelial growth factor production | IL1A, IL6, IL1B | 3 | 5.35714286 | 0.03095775 |
| 91 | BP | GO:0001774~microglial cell activation | IFNG, ITGB2, TNF | 3 | 5.35714286 | 0.03095775 |
| 92 | BP | GO:0043066~negative regulation of apoptotic process | PDGFRB, IL6, FAS, TIMP1, MPO, MMP9, IL2 | 7 | 12.5 | 0.03182766 |
| 93 | BP | GO:0007159~leukocyte cell-cell adhesion | ITGB2, ITGAL, ICAM1 | 3 | 5.35714286 | 0.0343176 |
| 94 | BP | GO:0002230~positive regulation of defense response to virus by host | STAT1, IL27, PTPN22 | 3 | 5.35714286 | 0.03821319 |
| 95 | BP | GO:0051091~positive regulation of sequence-specific DNA binding transcription factor activity | IL6, IL1B, TRIM21, TNF | 4 | 7.14285714 | 0.04110645 |
| 96 | BP | GO:0032743~positive regulation of interleukin-2 production | IL1A, CD4, IL1B | 3 | 5.35714286 | 0.04175709 |
| 97 | BP | GO:2001240~negative regulation of extrinsic apoptotic signaling pathway in absence of ligand | IL1A, IL1B, TNF | 3 | 5.35714286 | 0.04536546 |
| 98 | BP | GO:0034614~cellular response to reactive oxygen species | PDGFRA, MMP2, MMP9 | 3 | 5.35714286 | 0.04536546 |
| 99 | BP | GO:0033627~cell adhesion mediated by integrin | ITGB2, ITGAL, ICAM1 | 3 | 5.35714286 | 0.04879795 |
| 100 | BP | GO:0030335~positive regulation of cell migration | PDGFRB, PDGFRA, IL1B, MMP2, MMP9 | 5 | 8.92857143 | 0.0510655 |
| 101 | BP | GO:0006953~acute-phase response | CRP, IL22, IL6 | 3 | 5.35714286 | 0.0510655 |
| 102 | BP | GO:0007566~embryo implantation | IL1B, MMP2, MMP9 | 3 | 5.35714286 | 0.0510655 |
| 103 | BP | GO:0007259~JAK-STAT cascade | SOCS1, IFNG, STAT1 | 3 | 5.35714286 | 0.05234918 |
| 104 | BP | GO:0030217~T cell differentiation | CD4, PTPN22, IL2 | 3 | 5.35714286 | 0.05234918 |
| 105 | BP | GO:0050766~positive regulation of phagocytosis | IFNG, IL1B, TNF | 3 | 5.35714286 | 0.06734745 |
| 106 | BP | GO:0042542~response to hydrogen peroxide | PDGFRB, STAT1, MMP2 | 3 | 5.35714286 | 0.07461739 |
| 107 | BP | GO:0033209~tumor necrosis factor-mediated signaling pathway | STAT1, FAS, TNF | 3 | 5.35714286 | 0.0793458 |
| 108 | BP | GO:1904707~positive regulation of vascular smooth muscle cell proliferation | MMP2, TNF, MMP9 | 3 | 5.35714286 | 0.08134374 |
| 109 | BP | GO:0014823~response to activity | IL6, MMP2, TNF | 3 | 5.35714286 | 0.0829805 |
| 110 | BP | GO:0032757~positive regulation of interleukin-8 production | IL6, IL1B, TNF | 3 | 5.35714286 | 0.099467 |
| 111 | BP | GO:0045893~positive regulation of transcription, DNA-templated | IL6, CD4, STAT1, IL1B, TRIM21, TNF, HLA-DRB1 | 7 | 12.5 | 0.09981999 |
| 112 | BP | GO:0045893~positive regulation of transcription, DNA-templated | IL6, CD4, STAT1, IL1B, TRIM21, TNF, HLA-DRB1 | 7 | 12.5 | 0.09981999 |
| 113 | BP | GO:0001819~positive regulation of cytokine production | IL1A, IFNG, TNF | 3 | 5.35714286 | 0.1006006 |
| 114 | BP | GO:0051384~response to glucocorticoid | IL22, IL6, TNF | 3 | 5.35714286 | 0.10173102 |
| 115 | BP | GO:0009612~response to mechanical stimulus | STAT1, MMP2, MPO | 3 | 5.35714286 | 0.10173102 |
| 116 | BP | GO:0043627~response to estrogen | PDGFRB, KRT19, MMP2 | 3 | 5.35714286 | 0.10664839 |
| 117 | BP | GO:0006909~phagocytosis | ITGB2, PRTN3, ITGAL | 3 | 5.35714286 | 0.10866565 |
| 118 | BP | GO:0010508~positive regulation of autophagy | IFNG, MEFV, TRIM21 | 3 | 5.35714286 | 0.12274818 |
| 119 | BP | GO:0006952~defense response | STAT1, HLA-B, MPO | 3 | 5.35714286 | 0.12781728 |
| 120 | BP | GO:0043406~positive regulation of MAP kinase activity | PDGFRB, IL1B, TNF | 3 | 5.35714286 | 0.12983692 |
| 121 | BP | GO:0071347~cellular response to interleukin-1 | MMP2, IL17A, ICAM1 | 3 | 5.35714286 | 0.15214436 |
| 122 | BP | GO:0050829~defense response to Gram-negative bacterium | IL6, CD4, IL17A | 3 | 5.35714286 | 0.16249513 |
| 123 | BP | GO:0050731~positive regulation of peptidyl-tyrosine phosphorylation | IL6, CD4, ICAM1 | 3 | 5.35714286 | 0.16450198 |
| 124 | BP | GO:0046718~viral entry into host cell | ACE2, CD4, ICAM1 | 3 | 5.35714286 | 0.18221175 |
| 125 | BP | GO:0045944~positive regulation of transcription from RNA polymerase II promoter | IL1A, IL6, STAT1, IL1B, IL18, TNF, IL2, IL17A | 8 | 14.2857143 | 0.2476139 |
| 126 | BP | GO:0045944~positive regulation of transcription from RNA polymerase II promoter | IL1A, IL6, STAT1, IL1B, IL18, TNF, IL2, IL17A | 8 | 14.2857143 | 0.2476139 |
| 127 | BP | GO:0000165~MAPK cascade | IL1B, IL18, TNF | 3 | 5.35714286 | 0.25075069 |
| 128 | BP | GO:0007166~cell surface receptor signaling pathway | CD4, IFNG, CD3G, MS4A1 | 4 | 7.14285714 | 0.26233292 |
| 129 | BP | GO:0007169~transmembrane receptor protein tyrosine kinase signaling pathway | PDGFRB, PDGFRA, CD4 | 3 | 5.35714286 | 0.26289619 |
| 130 | BP | GO:0045471~response to ethanol | TNF, IL2, ICAM1 | 3 | 5.35714286 | 0.27012596 |
| 131 | BP | GO:0010629~negative regulation of gene expression | IFNG, PTPN22, MMP8, TNF | 4 | 7.14285714 | 0.27715922 |
| 132 | BP | GO:0018108~peptidyl-tyrosine phosphorylation | PDGFRB, PDGFRA, CD4 | 3 | 5.35714286 | 0.2864764 |
| 133 | BP | GO:0007155~cell adhesion | CD4, ITGB2, ITGAL, IL2, ICAM1 | 5 | 8.92857143 | 0.31352338 |
| 134 | BP | GO:0045766~positive regulation of angiogenesis | IL1A, IL1B, ITGB2 | 3 | 5.35714286 | 0.33909719 |
| 135 | BP | GO:0001666~response to hypoxia | IL1A, MMP2, TNF | 3 | 5.35714286 | 0.38935236 |
| 136 | BP | GO:0098609~cell-cell adhesion | ITGB2, ITGAL, ICAM1 | 3 | 5.35714286 | 0.40832833 |
| 137 | BP | GO:0051607~defense response to virus | IL6, IFNG, STAT1 | 3 | 5.35714286 | 0.50622269 |
| 138 | BP | GO:0009410~response to xenobiotic stimulus | STAT1, MMP2, TNF | 3 | 5.35714286 | 0.52598953 |
| 139 | BP | GO:0001525~angiogenesis | PDGFRB, MMP2, IL18 | 3 | 5.35714286 | 0.55045178 |
| 140 | BP | GO:0016477~cell migration | PDGFRB, ITGB2, MMP9 | 3 | 5.35714286 | 0.59709138 |
| 141 | BP | GO:0008285~negative regulation of cell proliferation | IL1A, IL6, IL1B | 3 | 5.35714286 | 0.90211346 |
| 142 | BP | GO:0016567~protein ubiquitination | SOCS1, MEFV, TRIM21 | 3 | 5.35714286 | 0.90211346 |
| 143 | BP | GO:0000122~negative regulation of transcription from RNA polymerase II promoter | IFNG, STAT1, FASLG, TNF | 4 | 7.14285714 | 0.90211346 |
| 144 | CC | GO:0005615~extracellular space | CRP, FCGBP, IL22, FASLG, IL27, MPO, TNF, ICAM1, THBD, TIMP1, MMP2, MMP3, IL18, MMP8, MMP9, MMP10, IL2, ACE2, IL1A, IL6, MMP13, IFNG, IL1B, PRTN3, MS4A1, HLA-DRB1, IL17A | 27 | 48.2142857 | 2.60E-11 |
| 145 | CC | GO:0009897~external side of plasma membrane | PDGFRA, ITGB2, FASLG, CD3G, ITGAL, TNF, ICAM1, THBD, CD4, FAS, CTLA4, MS4A1, HLA-DRB1, HLA-DQB1, IL17A | 15 | 26.7857143 | 8.02E-10 |
| 146 | CC | GO:0042613~MHC class II protein complex | HLA-DRB4, HLA-B, HLA-C, HLA-A, HLA-DRB1, HLA-DQA1, HLA-DQB1 | 7 | 12.5 | 1.90E-09 |
| 147 | CC | GO:0071556~integral component of lumenal side of endoplasmic reticulum membrane | HLA-DRB4, HLA-B, HLA-C, HLA-A, HLA-DRB1, HLA-DQA1, HLA-DQB1 | 7 | 12.5 | 2.92E-09 |
| 148 | CC | GO:0009986~cell surface | PDGFRB, ITGB2, HLA-B, HLA-C, IL27, HLA-A, ITGAL, TNF, ICAM1, IL1A, ACE2, THBD, FAS, MS4A1, HLA-DRB1 | 15 | 26.7857143 | 1.55E-08 |
| 149 | CC | GO:0005576~extracellular region | CRP, IL22, MMP1, MMP2, MMP3, IL18, GZMB, FASLG, IL27, MMP8, MPO, TNF, MMP9, IL2, MMP10, IL1A, ACE2, IL6, MMP13, IFNG, IL1B, PRTN3, TIMP1, IL17A | 24 | 42.8571429 | 1.55E-08 |
| 150 | CC | GO:0012507~ER to Golgi transport vesicle membrane | HLA-DRB4, HLA-B, HLA-C, HLA-A, HLA-DRB1, HLA-DQA1, HLA-DQB1 | 7 | 12.5 | 1.20E-07 |
| 151 | CC | GO:0030669~clathrin-coated endocytic vesicle membrane | CD4, HLA-DRB4, CD3G, HLA-DRB1, HLA-DQA1, HLA-DQB1 | 6 | 10.7142857 | 1.44E-05 |
| 152 | CC | GO:0031012~extracellular matrix | FCGBP, MMP13, MMP1, MMP2, MMP3, TIMP1, MMP8, MMP10 | 8 | 14.2857143 | 5.90E-05 |
| 153 | CC | GO:0005887~integral component of plasma membrane | PDGFRB, PDGFRA, HLA-B, HLA-C, FASLG, CD3G, HLA-A, TNF, ICAM1, THBD, CD4, CTLA4, MS4A1, HLA-DQA1, HLA-DRB1 | 15 | 26.7857143 | 1.36E-04 |
| 154 | CC | GO:0030666~endocytic vesicle membrane | ACE2, HLA-DRB4, HLA-DRB1, HLA-DQA1, HLA-DQB1 | 5 | 8.92857143 | 3.45E-04 |
| 155 | CC | GO:0070062~extracellular exosome | FCGBP, ITGB2, HLA-B, HLA-C, FASLG, HLA-A, ITGAL, MPO, MMP9, ICAM1, ACE2, KRT19, FAS, PRTN3, TIMP1, MS4A1, HLA-DRB1 | 17 | 30.3571429 | 0.00106763 |
| 156 | CC | GO:0030658~transport vesicle membrane | HLA-DRB4, HLA-DRB1, HLA-DQA1, HLA-DQB1 | 4 | 7.14285714 | 0.00137008 |
| 157 | CC | GO:0042612~MHC class I protein complex | HLA-B, HLA-C, HLA-A | 3 | 5.35714286 | 0.00180319 |
| 158 | CC | GO:0005765~lysosomal membrane | HLA-DRB4, HLA-B, HLA-A, CD68, HLA-DRB1, HLA-DQA1, HLA-DQB1 | 7 | 12.5 | 0.00312676 |
| 159 | CC | GO:0010008~endosome membrane | HLA-DRB4, HLA-B, HLA-A, HLA-DRB1, HLA-DQA1, HLA-DQB1 | 6 | 10.7142857 | 0.00367981 |
| 160 | CC | GO:0005886~plasma membrane | ITGB2, CD3G, FASLG, ITGAL, TNF, ICAM1, THBD, CTLA4, HLA-DQA1, PDGFRB, PDGFRA, HLA-DRB4, MMP2, HLA-B, HLA-C, HLA-A, ACE2, IL1A, CD4, KRT19, FAS, PRTN3, CD68, MS4A1, HLA-DRB1, HLA-DQB1 | 26 | 46.4285714 | 0.00556729 |
| 161 | CC | GO:0032588~trans-Golgi network membrane | HLA-DRB4, HLA-DRB1, HLA-DQA1, HLA-DQB1 | 4 | 7.14285714 | 0.01463763 |
| 162 | CC | GO:0044853~plasma membrane raft | ITGB2, PRTN3, MS4A1 | 3 | 5.35714286 | 0.01985319 |
| 163 | CC | GO:0045121~membrane raft | ACE2, CD4, FAS, TNF, ICAM1 | 5 | 8.92857143 | 0.02287756 |
| 164 | CC | GO:0001772~immunological synapse | GZMB, HLA-DRB1, ICAM1 | 3 | 5.35714286 | 0.02970513 |
| 165 | CC | GO:0005789~endoplasmic reticulum membrane | PDGFRA, CD4, HLA-DRB4, HLA-B, HLA-C, HLA-A, HLA-DRB1, HLA-DQA1, HLA-DQB1 | 9 | 16.0714286 | 0.03491419 |
| 166 | CC | GO:0000139~Golgi membrane | HLA-DRB4, HLA-B, HLA-C, HLA-A, HLA-DRB1, HLA-DQA1, HLA-DQB1 | 7 | 12.5 | 0.03491419 |
| 167 | CC | GO:0005788~endoplasmic reticulum lumen | ACE2, IL6, CD4, IL27, TIMP1 | 5 | 8.92857143 | 0.03552204 |
| 168 | CC | GO:0030670~phagocytic vesicle membrane | HLA-B, HLA-C, HLA-A | 3 | 5.35714286 | 0.06533562 |
| 169 | CC | GO:0005581~collagen trimer | MMP1, TIMP1, MMP8 | 3 | 5.35714286 | 0.09463319 |
| 170 | CC | GO:0016020~membrane | PDGFRB, PDGFRA, ITGB2, HLA-B, HLA-C, GZMB, HLA-A, ITGAL, TNF, ICAM1, CD4, FAS, MT-CO2, CD68, HLA-DQA1, HLA-DRB1, HLA-DQB1 | 17 | 30.3571429 | 0.10872373 |
| 171 | CC | GO:0055038~recycling endosome membrane | HLA-B, HLA-C, HLA-A | 3 | 5.35714286 | 0.10872373 |
| 172 | CC | GO:0016021~integral component of membrane | PDGFRB, HLA-DRB4, MT-CO1, HLA-B, HLA-C, FASLG, CD3G, HLA-A, ITGAL, TNF, ICAM1, ACE2, CD4, MMP13, FAS, CTLA4, MT-CO2, CD68, HLA-DQA1, HLA-DRB1, HLA-DQB1 | 21 | 37.5 | 0.17070983 |
| 173 | CC | GO:0031902~late endosome membrane | HLA-DRB4, CD68, HLA-DRB1 | 3 | 5.35714286 | 0.17070983 |
| 174 | CC | GO:0031410~cytoplasmic vesicle | PDGFRB, SOCS1, MEFV, TRIM21 | 4 | 7.14285714 | 0.17070983 |
| 175 | CC | GO:0005794~Golgi apparatus | PDGFRB, PDGFRA, HLA-B, HLA-C, CTLA4, HLA-A, HLA-DQB1 | 7 | 12.5 | 0.2148974 |
| 176 | CC | GO:1990904~ribonucleoprotein complex | TIA1, SSB, TRIM21 | 3 | 5.35714286 | 0.2244075 |
| 177 | CC | GO:0031901~early endosome membrane | HLA-B, HLA-C, HLA-A | 3 | 5.35714286 | 0.23322914 |
| 178 | CC | GO:0043235~receptor complex | PDGFRB, PDGFRA, ITGB2 | 3 | 5.35714286 | 0.28173269 |
| 179 | CC | GO:0005764~lysosome | IL1B, CD68, MPO | 3 | 5.35714286 | 0.46042597 |
| 180 | CC | GO:0005925~focal adhesion | PDGFRB, ITGB2, ICAM1 | 3 | 5.35714286 | 0.61989009 |
| 181 | CC | GO:0048471~perinuclear region of cytoplasm | STAT1, CTLA4, FASLG, PTPN22 | 4 | 7.14285714 | 0.64588001 |
| 182 | CC | GO:0005783~endoplasmic reticulum | HLA-B, HLA-C, HLA-A | 3 | 5.35714286 | 0.8203125 |
| 183 | CC | GO:0043231~intracellular membrane-bounded organelle | PDGFRB, GZMB, PRTN3, MPO | 4 | 7.14285714 | 0.8203125 |
| 184 | CC | GO:0005829~cytosol | TIA1, STAT1, UROS, IL18, GZMB, PTPN22, CD3G, IL27, MEFV, IL1A, KRT19, SOCS1, IL1B, FAS, PRTN3, TRIM21 | 16 | 28.5714286 | 0.8203125 |
| 185 | CC | GO:0005739~mitochondrion | MT-CO1, MMP2, UROS, GZMB, MT-CO2 | 5 | 8.92857143 | 0.8203125 |
| 186 | CC | GO:0032991~macromolecular complex | PDGFRA, STAT1, TNF | 3 | 5.35714286 | 0.8203125 |
| 187 | CC | GO:0005634~nucleus | PDGFRB, PDGFRA, TIA1, SSB, STAT1, MMP2, GZMB, FASLG, PTPN22, MEFV, MPO, IL1A, TRIM21 | 13 | 23.2142857 | 0.90210171 |
| 188 | CC | GO:0005737~cytoplasm | PDGFRB, PDGFRA, ACE2, TIA1, SSB, SOCS1, STAT1, IL18, GZMB, PTPN22, MEFV, TRIM21 | 12 | 21.4285714 | 0.90275515 |
| 189 | CC | GO:0005654~nucleoplasm | PDGFRA, TIA1, SOCS1, STAT1, MEFV, MPO, TRIM21, MS4A1 | 8 | 14.2857143 | 0.91657761 |
| 190 | MF | GO:0004252~serine-type endopeptidase activity | MMP13, MMP1, MMP2, MMP3, GZMB, PRTN3, MMP8, MMP9, MMP10 | 9 | 16.0714286 | 2.25E-06 |
| 191 | MF | GO:0004175~endopeptidase activity | ACE2, MMP13, MMP1, MMP2, MMP3, MMP8, MMP9 | 7 | 12.5 | 4.38E-06 |
| 192 | MF | GO:0004222~metalloendopeptidase activity | MMP13, MMP1, MMP2, MMP3, MMP8, MMP9, MMP10 | 7 | 12.5 | 3.92E-05 |
| 193 | MF | GO:0008270~zinc ion binding | ACE2, CD4, MMP13, MMP1, MMP2, MMP3, TIMP1, MEFV, MMP8, TRIM21, MMP9, MMP10 | 12 | 21.4285714 | 4.25E-04 |
| 194 | MF | GO:0008237~metallopeptidase activity | ACE2, MMP2, MMP3, MMP9 | 4 | 7.14285714 | 0.0120912 |
| 195 | MF | GO:0008233~peptidase activity | MMP1, MMP3, MMP8, MMP9 | 4 | 7.14285714 | 0.02809186 |
| 196 | MF | GO:0005125~cytokine activity | IL1A, IL22, IL6, IFNG, IL1B, IL18, FASLG, IL27, TIMP1, TNF, IL2, IL17A | 12 | 21.4285714 | 7.38E-10 |
| 197 | MF | GO:0023026~MHC class II protein complex binding | CD4, HLA-DRB4, MS4A1, HLA-DRB1, HLA-DQA1, HLA-DQB1 | 6 | 10.7142857 | 7.68E-07 |
| 198 | MF | GO:0042605~peptide antigen binding | HLA-B, HLA-C, HLA-A, HLA-DRB1, HLA-DQA1, HLA-DQB1 | 6 | 10.7142857 | 2.25E-06 |
| 199 | MF | GO:0032395~MHC class II receptor activity | HLA-C, HLA-DRB1, HLA-DQA1, HLA-DQB1 | 4 | 7.14285714 | 1.12E-04 |
| 200 | MF | GO:0046977~TAP binding | HLA-B, HLA-C, HLA-A | 3 | 5.35714286 | 3.81E-04 |
| 201 | MF | GO:0005515~protein binding | FCGBP, IL22, MT-CO1, ITGB2, CD3G, PTPN22, FASLG, IL27, ITGAL, MPO, TNF, ICAM1, THBD, CTLA4, TIMP1, TRIM21, PDGFRB, PDGFRA, MMP2, MMP3, IL18, HLA-B, HLA-C, HLA-A, MMP9, ACE2, IL1A, IFNG, IL1B, PRTN3, MT-CO2, MS4A1, HLA-DQB1, CRP, MEFV, SOCS1, HLA-DQA1, TIA1, SSB, STAT1, GZMB, IL2, CD4, KRT19, IL6, FAS, CD68, HLA-DRB1, IL17A | 49 | 87.5 | 0.00211595 |
| 202 | MF | GO:0042608~T cell receptor binding | CD3G, HLA-A, HLA-DRB1 | 3 | 5.35714286 | 0.00500043 |
| 203 | MF | GO:0004888~transmembrane signaling receptor activity | THBD, CD4, FAS, CD3G, ICAM1 | 5 | 8.92857143 | 0.02022953 |
| 204 | MF | GO:0005164~tumor necrosis factor receptor binding | STAT1, FASLG, TNF | 3 | 5.35714286 | 0.03182364 |
| 205 | MF | GO:0038023~signaling receptor activity | THBD, CD4, ITGB2, FAS, ICAM1 | 5 | 8.92857143 | 0.03378376 |
| 206 | MF | GO:0005102~receptor binding | PDGFRB, HLA-B, FASLG, PRTN3, IL27, HLA-A | 6 | 10.7142857 | 0.04043645 |
| 207 | MF | GO:0005178~integrin binding | IL1B, ITGB2, ITGAL, ICAM1 | 4 | 7.14285714 | 0.07313675 |
| 208 | MF | GO:0001618~virus receptor activity | ACE2, CD4, ICAM1 | 3 | 5.35714286 | 0.12356839 |
| 209 | MF | GO:0042802~identical protein binding | CRP, ACE2, CD4, STAT1, FAS, CD3G, MEFV, TRIM21, MS4A1, TNF, MMP9 | 11 | 19.6428571 | 0.12970978 |
| 210 | MF | GO:0019901~protein kinase binding | PDGFRB, CD4, SOCS1, ITGB2, TRIM21 | 5 | 8.92857143 | 0.2720343 |
| 211 | MF | GO:0008083~growth factor activity | IL6, TIMP1, IL2 | 3 | 5.35714286 | 0.37363576 |
| 212 | MF | GO:0019899~enzyme binding | PDGFRB, CD4, STAT1, PRTN3 | 4 | 7.14285714 | 0.42547104 |
| 213 | MF | GO:0044877~macromolecular complex binding | PDGFRA, KRT19, ITGB2, ITGAL | 4 | 7.14285714 | 0.47154822 |
| 214 | MF | GO:0042803~protein homodimerization activity | PDGFRA, CD4, STAT1, TRIM21, IL17A | 5 | 8.92857143 | 0.56421328 |
| 215 | MF | GO:0005509~calcium ion binding | CRP, THBD, MMP13 | 3 | 5.35714286 | 0.88194444 |
| 216 | MF | GO:0046872~metal ion binding | ACE2, MT-CO1, ITGB2, TIMP1, ITGAL, MPO, HLA-DQA1, HLA-DQB1 | 8 | 14.2857143 | 0.88194444 |
| 217 | MF | GO:0003723~RNA binding | TIA1, SSB, HLA-A, TRIM21 | 4 | 7.14285714 | 0.88194444 |
| 218 | MF | GO:0003677~DNA binding | SSB, STAT1, TRIM21 | 3 | 5.35714286 | 0.90270679 |
