# Supplementary material for: Identifying TNF and IL6 as potential hub genes and targeted drugs associated with scleritis: A bio-informative report
Source: Front Immunol. 2023 Mar 31;14:1098140. doi: 10.3389/fimmu.2023.1098140 (PMC10102337; doi:10.3389/fimmu.2023.1098140)
Supplement: Supplementary file 4 [file Table_4.docx]

**Supplementary Table S4** All significant pathways of a of KEGG database.

| Number | Term | Genes | Count | Gene Ratio (%) | FDR |
| --- | --- | --- | --- | --- | --- |
| 1 | hsa05332: Graft-versus-host disease | HLA-DRB4, HLA-B, HLA-C, GZMB, FASLG, HLA-A, TNF, IL2, IL1A, IL6, IFNG, IL1B, FAS, HLA-DQA1, HLA-DRB1, HLA-DQB1 | 16 | 28.5714286 | 1.31E-22 |
| 2 | hsa04940: Type I diabetes mellitus | HLA-DRB4, HLA-B, HLA-C, GZMB, FASLG, HLA-A, TNF, IL2, IL1A, IFNG, IL1B, FAS, HLA-DQA1, HLA-DRB1, HLA-DQB1 | 15 | 26.7857143 | 1.27E-20 |
| 3 | hsa05323: Rheumatoid arthritis | HLA-DRB4, MMP1, ITGB2, MMP3, IL18, ITGAL, TNF, ICAM1, IL1A, IL6, IFNG, IL1B, CTLA4, HLA-DQA1, HLA-DRB1, HLA-DQB1, IL17A | 17 | 30.3571429 | 5.44E-19 |
| 4 | hsa05330: Allograft rejection | HLA-DRB4, HLA-B, HLA-C, GZMB, FASLG, HLA-A, TNF, IL2, IFNG, FAS, HLA-DQA1, HLA-DRB1, HLA-DQB1 | 13 | 23.2142857 | 1.25E-17 |
| 5 | hsa05321: Inflammatory bowel disease | IL22, HLA-DRB4, STAT1, IL18, TNF, IL2, IL1A, IL6, IFNG, IL1B, HLA-DQA1, HLA-DRB1, HLA-DQB1, IL17A | 14 | 25 | 2.33E-16 |
| 6 | hsa05320: Autoimmune thyroid disease | HLA-DRB4, HLA-B, HLA-C, FAS, CTLA4, GZMB, FASLG, HLA-A, HLA-DRB1, IL2, HLA-DQA1, HLA-DQB1 | 12 | 21.4285714 | 5.09E-14 |
| 7 | hsa04659: Th17 cell differentiation | IL22, HLA-DRB4, STAT1, CD3G, IL27, IL2, IL6, CD4, IFNG, IL1B, HLA-DQA1, HLA-DRB1, HLA-DQB1, IL17A | 14 | 25 | 1.79E-13 |
| 8 | hsa05164: Influenza A | HLA-DRB4, STAT1, IL18, FASLG, TNF, ICAM1, IL1A, IL6, IFNG, IL1B, FAS, HLA-DQA1, HLA-DRB1, HLA-DQB1 | 14 | 25 | 6.40E-11 |
| 9 | hsa05166: Human T-cell leukemia virus 1 infection | HLA-DRB4, ITGB2, HLA-B, HLA-C, CD3G, HLA-A, ITGAL, TNF, IL2, ICAM1, IL6, CD4, HLA-DQA1, HLA-DRB1, HLA-DQB1 | 15 | 26.7857143 | 9.80E-11 |
| 10 | hsa05416: Viral myocarditis | HLA-DRB4, ITGB2, HLA-B, HLA-C, HLA-A, ITGAL, HLA-DRB1, HLA-DQA1, ICAM1, HLA-DQB1 | 10 | 17.8571429 | 2.67E-10 |
| 11 | hsa05169: Epstein-Barr virus infection | HLA-DRB4, STAT1, HLA-B, HLA-C, CD3G, HLA-A, ITGAL, TNF, ICAM1, IL6, FAS, HLA-DQA1, HLA-DRB1, HLA-DQB1 | 14 | 25 | 3.87E-10 |
| 12 | hsa04640: Hematopoietic cell lineage | IL1A, IL6, CD4, HLA-DRB4, IL1B, CD3G, MS4A1, TNF, HLA-DRB1, HLA-DQA1, HLA-DQB1 | 11 | 19.6428571 | 9.34E-10 |
| 13 | hsa05140: Leishmaniasis | IL1A, HLA-DRB4, IFNG, STAT1, IL1B, ITGB2, TNF, HLA-DRB1, HLA-DQA1, HLA-DQB1 | 10 | 17.8571429 | 2.09E-09 |
| 14 | hsa04612: Antigen processing and presentation | CD4, HLA-DRB4, IFNG, HLA-B, HLA-C, HLA-A, TNF, HLA-DRB1, HLA-DQA1, HLA-DQB1 | 10 | 17.8571429 | 2.18E-09 |
| 15 | hsa04514: Cell adhesion molecules | CD4, HLA-DRB4, ITGB2, HLA-B, HLA-C, CTLA4, HLA-A, ITGAL, HLA-DRB1, HLA-DQA1, ICAM1, HLA-DQB1 | 12 | 21.4285714 | 4.32E-09 |
| 16 | hsa05143: African trypanosomiasis | IL6, IFNG, IL1B, IL18, FAS, FASLG, TNF, ICAM1 | 8 | 14.2857143 | 5.46E-09 |
| 17 | hsa05152: Tuberculosis | IL1A, IL6, HLA-DRB4, IFNG, STAT1, IL1B, ITGB2, IL18, TNF, HLA-DRB1, HLA-DQA1, HLA-DQB1 | 12 | 21.4285714 | 1.64E-08 |
| 18 | hsa05144: Malaria | IL6, IFNG, IL1B, ITGB2, IL18, ITGAL, TNF, ICAM1 | 8 | 14.2857143 | 4.45E-08 |
| 19 | hsa04658: Th1 and Th2 cell differentiation | CD4, HLA-DRB4, IFNG, STAT1, CD3G, HLA-DRB1, IL2, HLA-DQA1, HLA-DQB1 | 9 | 16.0714286 | 1.61E-07 |
| 20 | hsa04060: Cytokine-cytokine receptor interaction | IL22, IL18, FASLG, IL27, TNF, IL2, IL1A, IL6, CD4, IFNG, IL1B, FAS, IL17A | 13 | 23.2142857 | 2.29E-07 |
| 21 | hsa05150: Staphylococcus aureus infection | KRT19, HLA-DRB4, ITGB2, ITGAL, HLA-DRB1, HLA-DQA1, ICAM1, HLA-DQB1 | 8 | 14.2857143 | 3.57E-06 |
| 22 | hsa04933: AGE-RAGE signaling pathway in diabetic complications | THBD, IL1A, IL6, STAT1, IL1B, MMP2, TNF, ICAM1 | 8 | 14.2857143 | 4.51E-06 |
| 23 | hsa05142: Chagas disease | IL6, IFNG, IL1B, FAS, FASLG, CD3G, TNF, IL2 | 8 | 14.2857143 | 4.94E-06 |
| 24 | hsa04145: Phagosome | HLA-DRB4, ITGB2, HLA-B, HLA-C, HLA-A, MPO, HLA-DRB1, HLA-DQA1, HLA-DQB1 | 9 | 16.0714286 | 5.98E-06 |
| 25 | hsa05145: Toxoplasmosis | HLA-DRB4, SOCS1, IFNG, STAT1, TNF, HLA-DRB1, HLA-DQA1, HLA-DQB1 | 8 | 14.2857143 | 8.25E-06 |
| 26 | hsa05417: Lipid and atherosclerosis | IL6, MMP1, IL1B, MMP3, IL18, FAS, FASLG, TNF, MMP9, ICAM1 | 10 | 17.8571429 | 8.25E-06 |
| 27 | hsa05168: Herpes simplex virus 1 infection | HLA-DRB4, STAT1, HLA-B, HLA-C, FASLG, HLA-A, TNF, IL6, IFNG, IL1B, FAS, HLA-DQA1, HLA-DRB1, HLA-DQB1 | 14 | 25 | 9.98E-06 |
| 28 | hsa04630: JAK-STAT signaling pathway | PDGFRB, PDGFRA, IL22, IL6, SOCS1, IFNG, STAT1, IL27, IL2 | 9 | 16.0714286 | 9.98E-06 |
| 29 | hsa04650: Natural killer cell mediated cytotoxicity | IFNG, ITGB2, FAS, GZMB, FASLG, ITGAL, TNF, ICAM1 | 8 | 14.2857143 | 1.63E-05 |
| 30 | hsa04672: Intestinal immune network for IgA production | IL6, HLA-DRB4, HLA-DRB1, IL2, HLA-DQA1, HLA-DQB1 | 6 | 10.7142857 | 1.92E-05 |
| 31 | hsa05322: Systemic lupus erythematosus | SSB, HLA-DRB4, IFNG, TRIM21, TNF, HLA-DRB1, HLA-DQA1, HLA-DQB1 | 8 | 14.2857143 | 2.53E-05 |
| 32 | hsa05418: Fluid shear stress and atherosclerosis | THBD, IL1A, IFNG, IL1B, MMP2, TNF, MMP9, ICAM1 | 8 | 14.2857143 | 2.75E-05 |
| 33 | hsa05162: Measles | IL1A, IL6, STAT1, IL1B, FAS, FASLG, CD3G, IL2 | 8 | 14.2857143 | 2.75E-05 |
| 34 | hsa04932: Non-alcoholic fatty liver disease | IL1A, IL6, MT-CO1, IL1B, FAS, MT-CO2, FASLG, TNF | 8 | 14.2857143 | 5.43E-05 |
| 35 | hsa05310: Asthma | HLA-DRB4, TNF, HLA-DRB1, HLA-DQA1, HLA-DQB1 | 5 | 8.92857143 | 5.61E-05 |
| 36 | hsa05163: Human cytomegalovirus infection | PDGFRA, IL6, IL1B, HLA-B, HLA-C, FAS, FASLG, HLA-A, TNF | 9 | 16.0714286 | 7.25E-05 |
| 37 | hsa05163: Human cytomegalovirus infection | PDGFRA, IL6, IL1B, HLA-B, HLA-C, FAS, FASLG, HLA-A, TNF | 9 | 16.0714286 | 7.25E-05 |
| 38 | hsa04668: TNF signaling pathway | IL6, IL1B, MMP3, FAS, TNF, MMP9, ICAM1 | 7 | 12.5 | 7.70E-05 |
| 39 | hsa05135: Yersinia infection | IL6, CD4, IL1B, IL18, MEFV, TNF, IL2 | 7 | 12.5 | 2.31E-04 |
| 40 | hsa05170: Human immunodeficiency virus 1 infection | CD4, HLA-B, HLA-C, FAS, FASLG, CD3G, HLA-A, TNF | 8 | 14.2857143 | 3.46E-04 |
| 41 | hsa04217: Necroptosis | IL1A, IFNG, STAT1, IL1B, FAS, FASLG, TNF | 7 | 12.5 | 4.97E-04 |
| 42 | hsa05134: Legionellosis | IL6, IL1B, ITGB2, IL18, TNF | 5 | 8.92857143 | 5.28E-04 |
| 43 | hsa05171: Coronavirus disease - COVID-19 | ACE2, IL6, STAT1, MMP1, IL1B, MMP3, TNF, IL2 | 8 | 14.2857143 | 5.46E-04 |
| 44 | hsa05200: Pathways in cancer | PDGFRB, PDGFRA, IL6, IFNG, STAT1, MMP1, MMP2, FAS, FASLG, MMP9, IL2 | 11 | 19.6428571 | 0.00117163 |
| 45 | hsa04380: Osteoclast differentiation | IL1A, SOCS1, IFNG, STAT1, IL1B, TNF | 6 | 10.7142857 | 0.00125379 |
| 46 | hsa05167: Kaposi sarcoma-associated herpesvirus infection | IL6, STAT1, HLA-B, HLA-C, FAS, HLA-A, ICAM1 | 7 | 12.5 | 0.00125379 |
| 47 | hsa05133: Pertussis | IL1A, IL6, IL1B, ITGB2, TNF | 5 | 8.92857143 | 0.00141987 |
| 48 | hsa04936: Alcoholic liver disease | IL6, IL1B, FAS, FASLG, TNF, IL17A | 6 | 10.7142857 | 0.00191879 |
| 49 | hsa05161: Hepatitis B | IL6, STAT1, FAS, FASLG, TNF, MMP9 | 6 | 10.7142857 | 0.00336703 |
| 50 | hsa05165: Human papillomavirus infection | PDGFRB, STAT1, HLA-B, HLA-C, FAS, FASLG, HLA-A, TNF | 8 | 14.2857143 | 0.00380442 |
| 51 | hsa05146: Amoebiasis | IL6, IFNG, IL1B, ITGB2, TNF | 5 | 8.92857143 | 0.00389482 |
| 52 | hsa04625: C-type lectin receptor signaling pathway | IL6, STAT1, IL1B, TNF, IL2 | 5 | 8.92857143 | 0.00410043 |
| 53 | hsa04621: NOD-like receptor signaling pathway | IL6, STAT1, IL1B, IL18, MEFV, TNF | 6 | 10.7142857 | 0.00540689 |
| 54 | hsa05130: Pathogenic Escherichia coli infection | IL6, IL1B, IL18, FAS, FASLG, TNF | 6 | 10.7142857 | 0.00696588 |
| 55 | hsa04010: MAPK signaling pathway | PDGFRB, IL1A, PDGFRA, IL1B, FAS, FASLG, TNF | 7 | 12.5 | 0.00835983 |
| 56 | hsa04218: Cellular senescence | IL1A, IL6, HLA-B, HLA-C, HLA-A | 5 | 8.92857143 | 0.01558443 |
| 57 | hsa05160: Hepatitis C | IFNG, STAT1, FAS, FASLG, TNF | 5 | 8.92857143 | 0.0156558 |
| 58 | hsa01523: Antifolate resistance | IL6, IL1B, TNF | 3 | 5.35714286 | 0.01597987 |
| 59 | hsa05022: Pathways of neurodegeneration - multiple diseases | IL1A, IL6, MT-CO1, IL1B, FAS, MT-CO2, FASLG, TNF | 8 | 14.2857143 | 0.02152288 |
| 60 | hsa05020: Prion disease | IL1A, IL6, MT-CO1, IL1B, MT-CO2, TNF | 6 | 10.7142857 | 0.0226081 |
| 61 | hsa04061: Viral protein interaction with cytokine and cytokine receptor | IL6, IL18, TNF, IL2 | 4 | 7.14285714 | 0.0226081 |
| 62 | hsa05010: Alzheimer disease | IL1A, IL6, MT-CO1, IL1B, FAS, MT-CO2, TNF | 7 | 12.5 | 0.02431306 |
| 63 | hsa04620: Toll-like receptor signaling pathway | IL6, STAT1, IL1B, TNF | 4 | 7.14285714 | 0.02431306 |
| 64 | hsa05205: Proteoglycans in cancer | MMP2, FAS, FASLG, TNF, MMP9 | 5 | 8.92857143 | 0.03174243 |
| 65 | hsa04210: Apoptosis | FAS, GZMB, FASLG, TNF | 4 | 7.14285714 | 0.04490792 |
