# Supplementary material for: Identifying TNF and IL6 as potential hub genes and targeted drugs associated with scleritis: A bio-informative report
Source: Front Immunol. 2023 Mar 31;14:1098140. doi: 10.3389/fimmu.2023.1098140 (PMC10102337; doi:10.3389/fimmu.2023.1098140)
Supplement: Supplementary file 5 [file Table_5.docx]

**Supplementary Table S5** Top ten hub genes obtained by five algorithms of Cytohubba.

|  | MNC | |  | Degree | |  | Closeness | |  | | EPC | |  | MCC | |  |
| --- | --- | --- | --- | --- | --- | --- | --- | --- | --- | --- | --- | --- | --- | --- | --- | --- |
|  | **Gene** | **Score** | | **Gene** | **Score** | | **Gene** | **Score** | |  | **Gene** | **Score** | | **Gene** | **Score** | |
| 1 | **TNF** | **46** | | **TNF** | **46** | | **TNF** | **48.5** | | **TNF** | | **14.408** | | **IL6** | **1490909447064** | |
| 2 | **IL6** | **40** | | **IL6** | **40** | | **IL6** | **45.66667** | | CD4 | | 13.969 | | IL1β | 1490909379240 | |
| 3 | CD4 | 39 | | CD4 | 39 | | CD4 | 45.33333 | | **IL6** | | **13.757** | | IL17A | 1490904596160 | |
| 4 | IL1β | 35 | | IL1β | 35 | | IL1β | 42.66667 | | IL2 | | 13.404 | | IFNγ | 1490900595840 | |
| 5 | IL2 | 35 | | L2 | 35 | | L2 | 42.66667 | | IL17A | | 13.313 | | ICAM1 | 1490868303120 | |
| 6 | IFNγ | 33 | | IFNγ | 33 | | IFNγ | 41.66667 | | IFNγ | | 13.108 | | IL18 | 1490777124480 | |
| 7 | IL17A | 32 | | IL17A | 32 | | IL17A | 41.16667 | | IL1β | | 12.616 | | **TNF** | **1490348232338** | |
| 8 | ICAM1 | 30 | | ICAM1 | 30 | | ICAM1 | 40.16667 | | CTLA4 | | 12.462 | | CD68 | 1489746867984 | |
| 9 | CTLA4 | 30 | | CTLA4 | 30 | | CTLA4 | 40.16667 | | ICAM1 | | 12.419 | | MMP9 | 1489268022720 | |
| 10 | MMP9 | 29 | | MMP9 | 29 | | MMP9 | 39.66667 | | MMP9 | | 12.317 | | CRP | 1488741817080 | |
