# Supplementary material for: Identifying TNF and IL6 as potential hub genes and targeted drugs associated with scleritis: A bio-informative report
Source: Front Immunol. 2023 Mar 31;14:1098140. doi: 10.3389/fimmu.2023.1098140 (PMC10102337; doi:10.3389/fimmu.2023.1098140)
Supplement: Supplementary file 6 [file Table_6.docx]

**Supplementary Table S6** The information of all identified drugs.

| **Number** | **Gene** | **Drug** | **Interaction Types** | **Sources** | **PMIDs** | **FDA Approved or Not** | **Query Score** | **Interaction Score** |
| --- | --- | --- | --- | --- | --- | --- | --- | --- |
| 1 | TNF | HYDROXYCHLOROQUINE | n/a | NCI | 9002011 | Yes | 21.52 | 4.55 |
| 2 | TNF | MIDAZOLAM | n/a | NCI | 16406030 | Yes | 14.63 | 1.55 |
| 3 | TNF | RIFAMPIN | n/a | PharmGKB | 22151084 | Yes | 13.16 | 1.39 |
| 4 | TNF | AFELIMOMAB | inhibitor | ChemblInteractions | 10829362 | No | 12.91 | 2.73 |
| 5 | TNF | INFLIXIMAB | inhibitor | TdgClinicalTrial/ChemblInteractions/TEND/PharmGKB/TTD | 16720636/16909270/23057546/16456024 | Yes | 11.78 | 1.24 |
| 6 | TNF | CEFOTAXIME | n/a | NCI | 8354907/10989981 | Yes | 8.61 | 1.82 |
| 7 | TNF | ONERCEPT | inhibitor | ChemblInteractions | None found | No | 8.61 | 1.82 |
| 8 | TNF | PEGSUNERCEPT | inhibitor | ChemblInteractions/TTD | None found | No | 8.61 | 1.82 |
| 9 | TNF | GEMCITABINE | n/a | PharmGKB | 31616045 | Yes | 8.61 | 1.82 |
| 10 | TNF | RISPERIDONE | n/a | NCI | 15567770/11545247 | Yes | 4.3 | 0.91 |
| 11 | TNF | ADALIMUMAB | antibody/inhibitor | TdgClinicalTrial/ChemblInteractions/TEND/PharmGKB/TTD | 12044041/16720636/16909270/23057546 | Yes | 4.3 | 0.91 |
| 12 | TNF | THALIDOMIDE | inhibitor | TdgClinicalTrial/TEND/TTD | 8755512/12046682/12167383/12105857 | Yes | 4.3 | 0.91 |
| 13 | TNF | AZ9773 | inhibitor | ChemblInteractions | None found | No | 4.3 | 0.91 |
| 14 | TNF | RABEPRAZOLE | n/a | NCI | 16815316 | Yes | 4.3 | 0.91 |
| 15 | TNF | ETANERCEPT | antibody/inhibitor | TdgClinicalTrial/ChemblInteractions/TEND/PharmGKB/TTD | 16720636/16909270/23057546/26244882 | Yes | 4.3 | 0.91 |
| 16 | TNF | METHIMAZOLE | n/a | NCI | 8491516 | Yes | 4.3 | 0.91 |
| 17 | TNF | CARBAMAZEPINE | n/a | NCI/PharmGKB | 15565432/11294926 | Yes | 4.3 | 0.91 |
| 18 | TNF | ABBV-257 | n/a | TTD | None found | No | 4.3 | 0.45 |
| 19 | TNF | VADIMEZAN | inducer | TALC | None found | No | 4.3 | 0.91 |
| 20 | TNF | DIGOXIN | n/a | DTC | None found | Yes | 2.87 | 0.61 |
| 21 | TNF | LACTULOSE | n/a | NCI | 11226652 | Yes | 2.15 | 0.45 |
| 22 | TNF | CERTOLIZUMAB PEGOL | inhibitor | ChemblInteractions | None found | Yes | 2.15 | 0.45 |
| 23 | TNF | ATORVASTATIN | n/a | PharmGKB | 18997459 | Yes | 2.15 | 0.45 |
| 24 | TNF | LENALIDOMIDE | n/a | ClearityFoundationClinicalTrial/TTD |  | Yes | 2.15 | 0.45 |
| 25 | TNF | ENBREL | n/a | TTD | None found | No | 2.15 | 0.45 |
| 26 | TNF | NERELIMOMAB | inhibitor | ChemblInteractions | None found | No | 1.72 | 0.36 |
| 27 | TNF | PLACULUMAB | inhibitor | TdgClinicalTrial/ChemblInteractions/TTD |  | No | 1.43 | 0.3 |
| 28 | TNF | HOMIDIUM BROMIDE | n/a | DTC | None found | No | 1.43 | 0.3 |
| 29 | TNF | METHYLENE BLUE | n/a | DTC | None found | Yes | 1.43 | 0.3 |
| 30 | TNF | MEROPENEM | n/a | NCI | 8354907 | Yes | 1.23 | 0.26 |
| 31 | TNF | ETHAMBUTOL | n/a | PharmGKB | 22151084 | Yes | 1.08 | 0.23 |
| 32 | TNF | CERTOLIZUMAB | n/a | TTD | None found | No | 1.08 | 0.23 |
| 33 | TNF | GOLIMUMAB | antibody/inhibitor | TdgClinicalTrial/ChemblInteractions/TEND/TTD | 21079302 | Yes | 1.08 | 0.23 |
| 34 | TNF | DERSALAZINE | n/a | TdgClinicalTrial | None found | No | 1.08 | 0.23 |
| 35 | TNF | PYRAZINAMIDE | n/a | PharmGKB | 22151084 | Yes | 1.08 | 0.23 |
| 36 | TNF | GENTAMICIN | n/a | NCI | 14565862 | Yes | 1.08 | 0.23 |
| 37 | TNF | LENERCEPT | inhibitor | ChemblInteractions | None found | No | 0.86 | 0.18 |
| 38 | TNF | ISONIAZID | n/a | PharmGKB | 22151084 | Yes | 0.86 | 0.18 |
| 39 | TNF | SOBLIDOTIN | n/a | NCI | 11855751 | No | 0.86 | 0.18 |
| 40 | TNF | PF-04236921 | n/a | TdgClinicalTrial | None found | No | 0.78 | 0.17 |
| 41 | TNF | OZORALIZUMAB | inhibitor | TdgClinicalTrial/ChemblInteractions | None found | No | 0.72 | 0.15 |
| 42 | TNF | CELASTROL | n/a | TTD | None found | No | 0.72 | 0.15 |
| 43 | TNF | CARBOPLATIN | n/a | PharmGKB | 31616045 | Yes | 0.72 | 0.15 |
| 44 | TNF | SORAFENIB | n/a | PharmGKB | 22736425 | Yes | 0.66 | 0.14 |
| 45 | TNF | BCG VACCINE | n/a | NCI | 10022737 | No | 0.61 | 0.13 |
| 46 | TNF | RUTIN | n/a | NCI | 12423426 | No | 0.61 | 0.13 |
| 47 | TNF | LAPACHONE | n/a | NCI | 10075082 | No | 0.61 | 0.13 |
| 48 | TNF | OMEPRAZOLE | n/a | NCI | 16815316 | Yes | 0.51 | 0.05 |
| 49 | TNF | BUPIVACAINE | n/a | NCI | 15781526 | Yes | 0.51 | 0.11 |
| 50 | TNF | INSULIN | n/a | NCI | 16125526/9287059 | Yes | 0.48 | 0.1 |
| 51 | TNF | PENTOXIFYLLINE | n/a | TTD | None found | Yes | 0.48 | 0.1 |
| 52 | TNF | HALOFUGINONE | n/a | NCI | 16769768 | No | 0.45 | 0.1 |
| 53 | TNF | PROPYLTHIOURACIL | n/a | NCI | 15119959 | Yes | 0.45 | 0.1 |
| 54 | TNF | CYCLOSPORINE | n/a | PharmGKB | 18444945 | Yes | 0.45 | 0.1 |
| 55 | TNF | STAVUDINE | n/a | PharmGKB | 20887379 | Yes | 0.41 | 0.09 |
| 56 | TNF | REMTOLUMAB | n/a | TTD | None found | No | 0.37 | 0.08 |
| 57 | TNF | PYRIDOXINE | n/a | NCI | 16277693 | Yes | 0.32 | 0.07 |
| 58 | TNF | MILTEFOSINE | n/a | NCI | 7883777 | Yes | 0.26 | 0.06 |
| 59 | TNF | ORTATAXEL | n/a | TTD | None found | No | 0.23 | 0.05 |
| 60 | TNF | DIDANOSINE | n/a | NCI | 9430255 | Yes | 0.23 | 0.05 |
| 61 | TNF | AMPHOTERICIN B | n/a | DTC | None found | Yes | 0.15 | 0.03 |
| 62 | TNF | SPIRONOLACTONE | n/a | NCI | 16837769 | Yes | 0.14 | 0.03 |
| 63 | TNF | LENABASUM | n/a | TTD | None found | No | 0.14 | 0.03 |
| 64 | TNF | NAFAMOSTAT | n/a | TTD | None found | No | 0.13 | 0.03 |
| 65 | TNF | BENZO[E]PYRENE | n/a | DTC | None found | No | 0.13 | 0.03 |
| 66 | TNF | ALTEPLASE | n/a | NCI | 8615653 | Yes | 0.11 | 0.02 |
| 67 | TNF | GLIMEPIRIDE | n/a | NCI | 14686960 | Yes | 0.11 | 0.02 |
| 68 | TNF | 5,7-DIHYDROXY-4-METHYLCOUMARIN | n/a | DTC | None found | No | 0.11 | 0.02 |
| 69 | IL6 | CLAZAKIZUMAB | inhibitor | TdgClinicalTrial/ChemblInteractions/TTD | None found | No | 17.22 | 9.89 |
| 70 | IL6 | RITUXIMAB | n/a | PharmGKB | 26384320 | Yes | 17.22 | 9.89 |
| 71 | IL6 | METRONIDAZOLE | n/a | NCI | 12111578 | Yes | 12.91 | 7.42 |
| 72 | IL6 | IFOSFAMIDE | n/a | NCI | 9260581 | Yes | 8.61 | 2.47 |
| 73 | IL6 | ECHINACEA, UNSPECIFIED | n/a | NCI | 9568541 | No | 4.3 | 2.47 |
| 74 | IL6 | OLOKIZUMAB | inhibitor | TdgClinicalTrial/ChemblInteractions/TTD | 24641941 | No | 4.3 | 2.47 |
| 75 | IL6 | SILTUXIMAB | antagonist/antibody/inhibitor | MyCancerGenome/ChemblInteractions/TTD | 8823310 | Yes | 4.3 | 2.47 |
| 76 | IL6 | LEVOFLOXACIN | n/a | NCI | 12714806 | Yes | 2.15 | 1.24 |
| 77 | IL6 | FENTANYL | n/a | NCI | 9527747 | Yes | 2.15 | 1.24 |
| 78 | IL6 | IBUDILAST | n/a | TTD | None found | No | 2.15 | 1.24 |
| 79 | IL6 | GEMFIBROZIL | n/a | NCI | 8941582 | Yes | 1.72 | 0.99 |
| 80 | IL6 | LINEZOLID | n/a | NCI | 14561977 | Yes | 1.23 | 0.71 |
| 81 | IL6 | INSULIN | n/a | NCI | 17392554 | Yes | 1.08 | 0.62 |
| 82 | IL6 | SIRUKUMAB | inhibitor | ChemblInteractions/TTD | None found | No | 1.01 | 0.29 |
| 83 | IL6 | ELSILIMOMAB | inhibitor | ChemblInteractions | None found | No | 0.96 | 0.55 |
| 84 | IL6 | PF-04236921 | inhibitor | ChemblInteractions/TTD | None found | No | 0.91 | 0.26 |
| 85 | IL6 | SAQUINAVIR | n/a | NCI | 15388451 | Yes | 0.86 | 0.49 |
| 86 | IL6 | RIBAVIRIN | n/a | PharmGKB | None found | Yes | 0.86 | 0.25 |
| 87 | IL6 | ADALIMUMAB | n/a | PharmGKB | 24253594 | Yes | 0.61 | 0.35 |
| 88 | IL6 | FENOFIBRATE | n/a | PharmGKB | 16607077 | Yes | 0.54 | 0.31 |
| 89 | IL6 | NELFINAVIR | n/a | NCI | 15388451 | Yes | 0.34 | 0.2 |
| 90 | IL6 | INFLIXIMAB | n/a | PharmGKB | 24253594 | Yes | 0.34 | 0.1 |
| 91 | IL6 | COR-001 | n/a | TTD | None found | No | 0.3 | 0.17 |
| 92 | IL6 | ETANERCEPT | n/a | PharmGKB | 24253594 | Yes | 0.13 | 0.08 |
| 93 | IL6 | CISPLATIN | n/a | CIViC | 21273582 | Yes | 0.06 | 0.03 |
| 94 | CD4 | ZANOLIMUMAB | n/a | Tdg Clinical Trial/ ChemblInteractions TTD | None found | No | 12.91 | 20.61 |
| 95 | CD4 | TREGALIZUMAB | agonist (activating), antibody (inhibitory) | TdgClinicalTrial/ChemblInteractions TTD | None found | No | 12.91 | 20.61 |
| 96 | CD4 | IBALIZUMAB | antagonist (inhibitory), antibody (inhibitory), inhibitor (inhibitory) | ChemblInteractions | None found | Yes | 8.61 | 13.74 |
| 97 | CD4 | PRILIXIMAB | negative modulator (inhibitory) | ChemblInteractions | None found | No | 4.3 | 6.87 |
| 98 | CD4 | CLENOLIXIMAB | inhibitor (inhibitory) | ChemblInteractions | None found | No | 4.3 | 6.87 |
| 99 | CD4 | TRX-1 | inhibitor (inhibitory) | ChemblInteractions | None found | No | 4.3 | 6.87 |
| 100 | CD4 | CEDELIZUMAB | inhibitor (inhibitory) | ChemblInteractions | None found | No | 4.3 | 6.87 |
| 101 | CD4 | KELIXIMAB | negative modulator (inhibitory) | ChemblInteractions | None found | No | 4.3 | 6.87 |
| 102 | CD4 | HERBIMYCIN | n/a | NCI | 1833207 | No | 4.3 | 6.87 |
| 103 | HLA-DQA1 | LUMIRACOXIB | n/a | PharmGKB | 20639878 | No | 2.15 | 7.73 |
| 104 | HLA-DQA1 | LAPATINIB | n/a | PharmGKB/ FDA | 24687830 21245432 | Yes | 0.64 | 2.29 |
| 105 | HLA-DQA1 | AZATHIOPRINE | n/a | PharmGKB | 25217962 | Yes | 0.34 | 1.24 |
| 106 | HLA-DQA1 | MERCAPTOPURINE | n/a | PharmGKB | 25217962 | Yes | 0.24 | 0.86 |
