# Supplementary material for: Identifying TNF and IL6 as potential hub genes and targeted drugs associated with scleritis: A bio-informative report
Source: Front Immunol. 2023 Mar 31;14:1098140. doi: 10.3389/fimmu.2023.1098140 (PMC10102337; doi:10.3389/fimmu.2023.1098140)
Supplement: Supplementary file 7 [file Table_7.docx]

**Supplementary Table S7-1** Top ten hub genes obtained by five algorithms of Cytohubba (Classification by etiology).

|  | Infection | Non-infection |
| --- | --- | --- |
| 1 | **TNF** | **TNF** |
| 2 | **HLA-DQA1** | **IL6** |
| 3 | HLA-DRB1 | CD4 |
| 4 | HLA-C | ICAM1 |
| 5 | HLA-DQB1 | MMP9 |
| 6 | ACE2 | IL18 |
| 7 |  | CTLA4 |
| 8 |  | CD68 |
| 9 |  | CRP |
| 10 |  | MMP2 |

**Supplementary Table S7-2** Top ten hub genes obtained by five algorithms of Cytohubba (Classification by location).

|  | Anterior | Posterior |
| --- | --- | --- |
| 1 | **TNF** | **TNF** |
| 2 | **IL6** | **CD4** |
| 3 | CTLA4 | CTLA4 |
| 4 | GZMB | IL6 |
| 5 | ICAM1 | PTPN22 |
| 6 | CD68 | PRTN3 |
| 7 | ITGAL | CRP |
| 8 | ITGB2 | HLA-A |
| 9 | MPO | HLA-B |
| 10 | FAS | MEFV |
