# Supplementary figures and images for: Identifying TNF and IL6 as potential hub genes and targeted drugs associated with scleritis: A bio-informative report
Source: Front Immunol. 2023 Mar 31;14:1098140. doi: 10.3389/fimmu.2023.1098140 (PMC10102337; doi:10.3389/fimmu.2023.1098140)

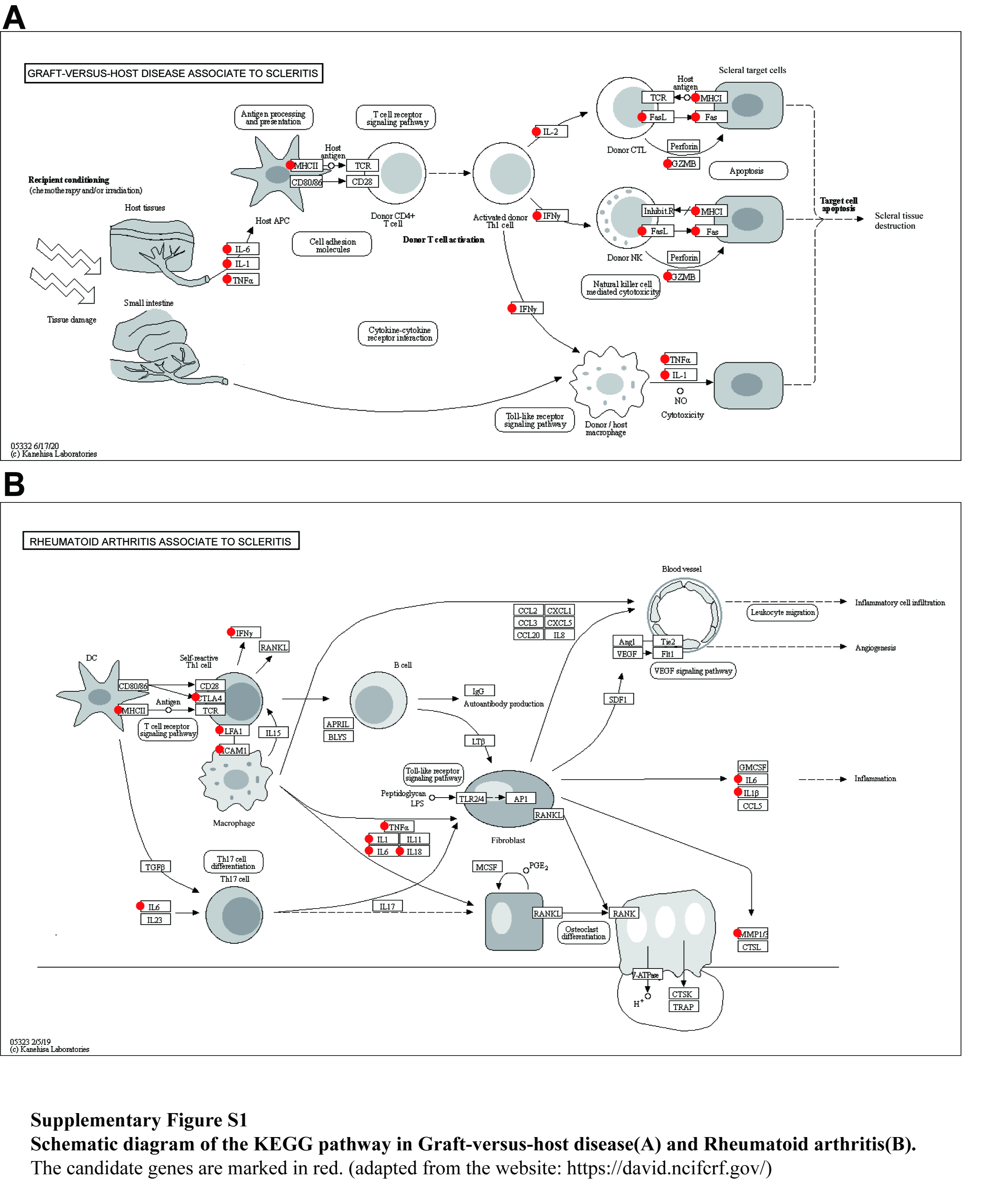

Supplement: Supplementary file 8 [file Image_1.tif]

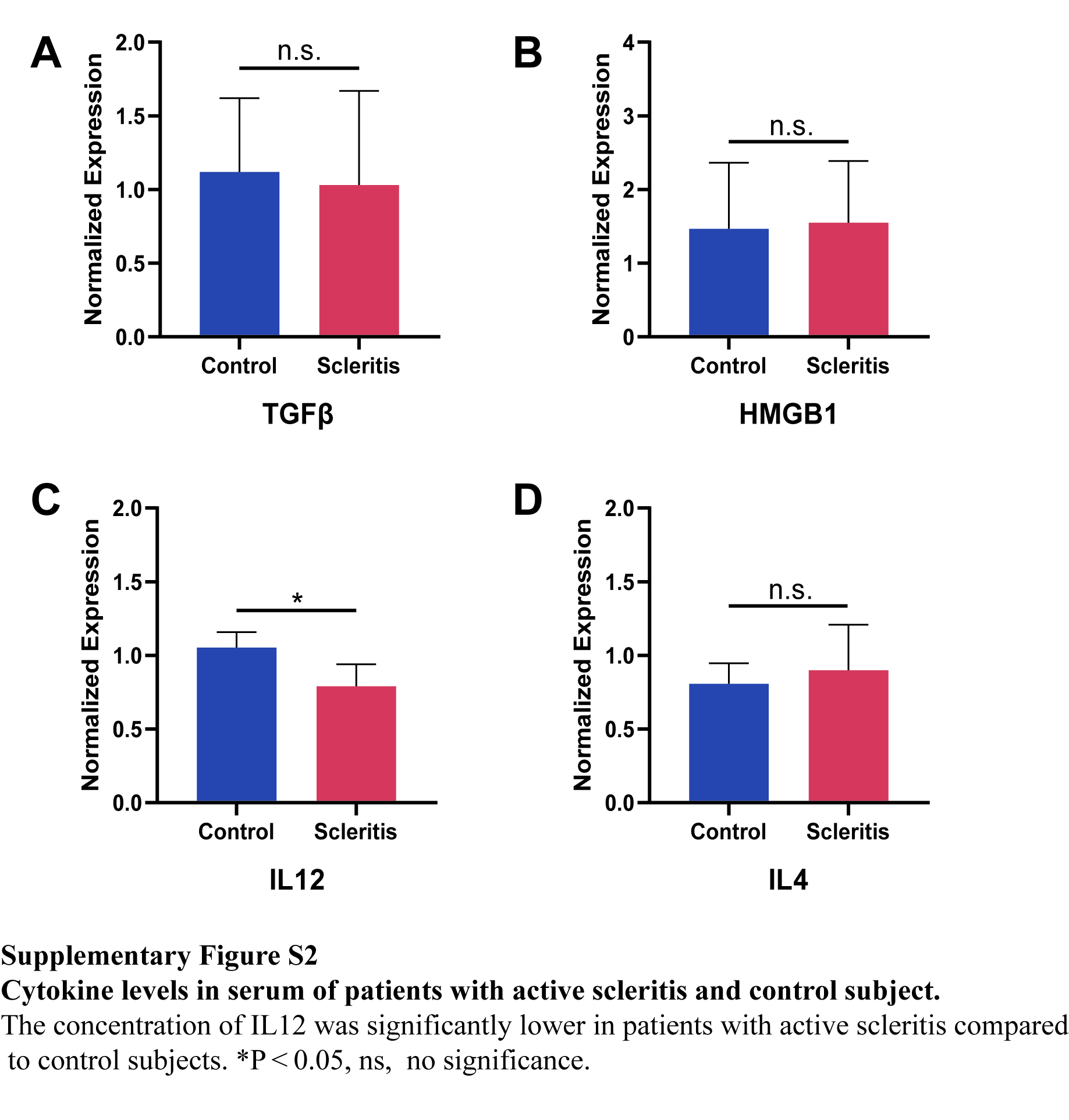

Supplement: Supplementary file 9 [file Image_2.tif]

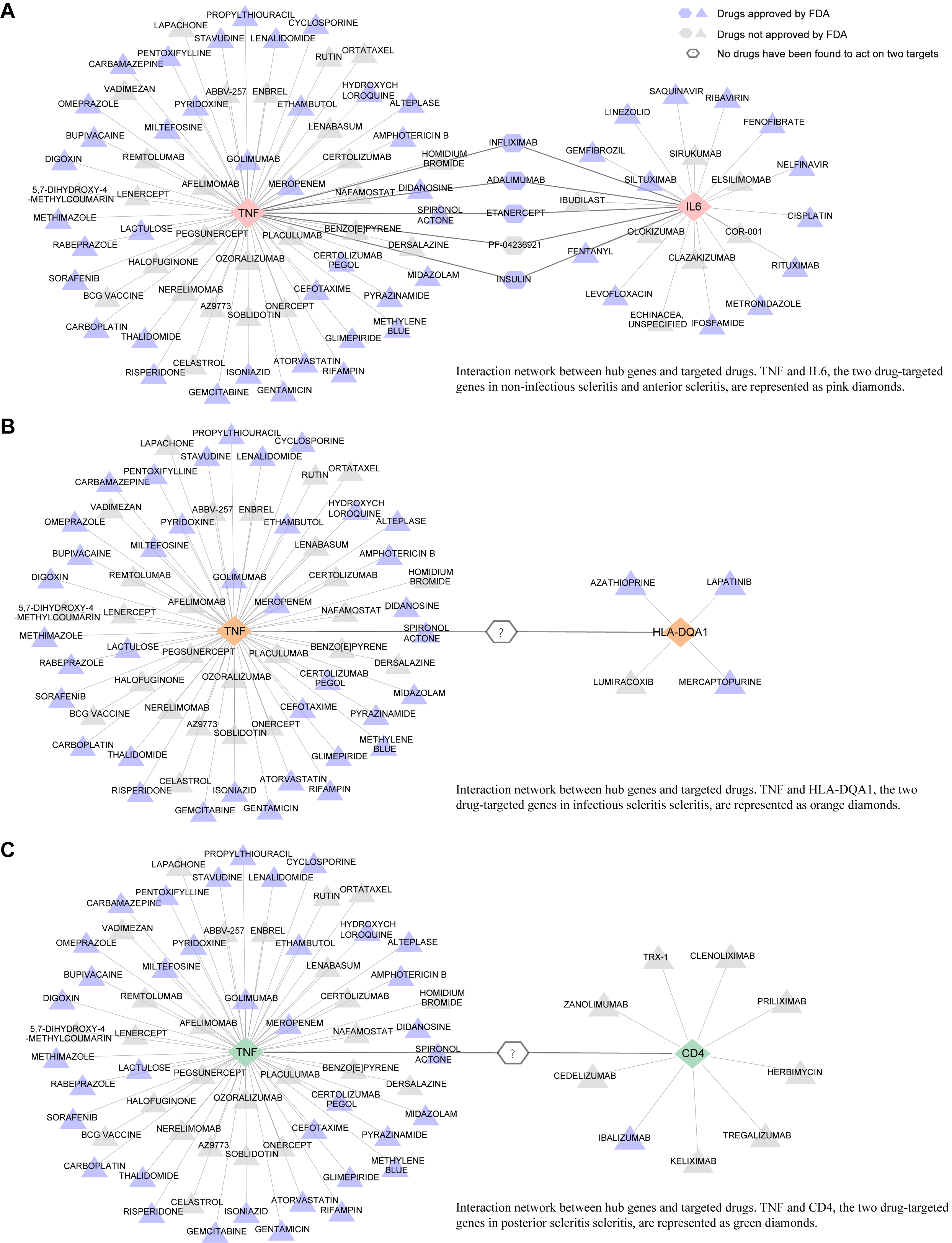

Supplement: Supplementary file 10 [file Image_3.tif]
